# The *CLV3* Homolog in *Setaria viridis* Selectively Controls Inflorescence Meristem Size

**DOI:** 10.3389/fpls.2021.636749

**Published:** 2021-02-15

**Authors:** Chuanmei Zhu, Lei Liu, Olivia Crowell, Hui Zhao, Thomas P. Brutnell, David Jackson, Elizabeth A. Kellogg

**Affiliations:** ^1^Donald Danforth Plant Science Center, St. Louis, MO, United States; ^2^Cold Spring Harbor Laboratory, Cold Spring Harbor, NY, United States; ^3^Institute of Tropical Bioscience and Biotechnology and Hainan Key Laboratory for Biosafety Monitoring and Molecular Breeding in Off-Season Reproduction Regions, Chinese Academy of Tropical Agricultural Sciences, Haikou, China; ^4^Joint Laboratory for Photosynthesis Enhancement and C_4_ Rice Development, Biotechnology Research Institute, Chinese Academy of Agricultural Sciences, Beijing, China

**Keywords:** inflorescence development, Setaria, FON2, CLE, CLV3/ESR-related, grass, meristem maintenance, spikelet

## Abstract

The CLAVATA pathway controls meristem size during inflorescence development in both eudicots and grasses, and is initiated by peptide ligands encoded by *CLV3*/*ESR*-related (*CLE*) genes. While CLV3 controls all shoot meristems in *Arabidopsis*, evidence from cereal grasses indicates that different meristem types are regulated by different CLE peptides. The rice peptide FON2 primarily controls the size of the floral meristem, whereas the orthologous peptides CLE7 and CLE14 in maize have their most dramatic effects on inflorescence and branch meristems, hinting at diversification among CLE responses in the grasses. *Setaria viridis* is more closely related to maize than to rice, so can be used to test whether the maize CLE network can be generalized to all members of subfamily Panicoideae. We used CRISPR-Cas9 in *S. viridis* to knock out the *SvFON2* gene, the closest homolog to *CLV3* and *FON2*. *Svfon2* mutants developed larger inflorescence meristems, as in maize, but had normal floral meristems, unlike *Osfon2*, suggesting a panicoid-specific CLE network. Vegetative traits such as plant height, tiller number and leaf number were not significantly different between mutant and wild type plants, but time to heading was shorter in the mutants. *In situ* hybridization showed strong expression of *Svfon2* in the inflorescence and branch meristems, consistent with the mutant phenotype. Using bioinformatic analysis, we predicted the co-expression network of *SvFON2* and its signaling components, which included genes known to control inflorescence architecture in maize as well as genes of unknown function. The similarity between SvFON2 function in Setaria and maize suggests that its developmental specialization in inflorescence meristem control may be shared among panicoid grasses.

## Introduction

The grass family (Poaceae) contains many agronomically important cereal crops, such as maize and rice, which have fed the world since the dawn of civilization. Their inflorescences, the flower- and seed-bearing structures, have been under constant selection for enhanced productivity during crop domestication. All inflorescence structures ultimately come from the inflorescence meristem, a group of pluripotent cells that is responsible for replenishing the stem cell pool as well as initiating lateral primordia. Therefore, understanding how the inflorescence meristem maintains a proper size is key to improving crop yields.

**Figure 1:**
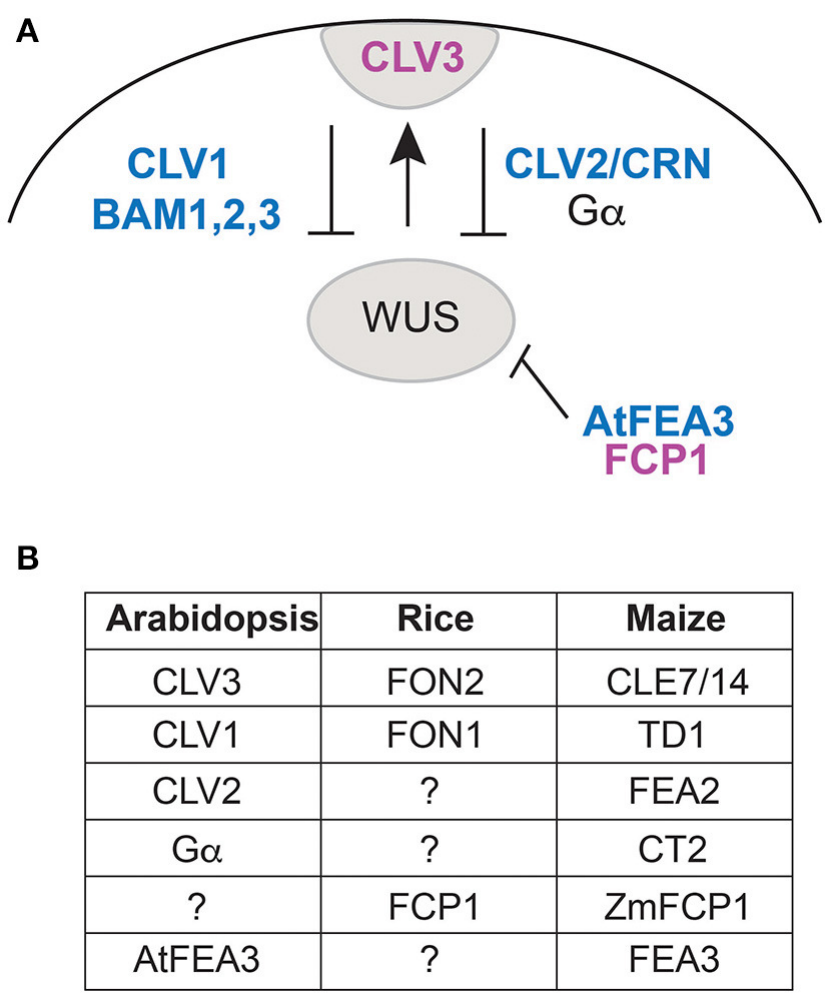
**(A)** Diagram of the proteins involved in maintenance of meristem size in Arabidopsis, focusing on those discussed in this paper (modified from Figure 2 in Kitagawa and Jackson, 2019). CLV3 is expressed at the apex of the meristem, where it interacts with CLV1 and BAM1-3, or with CLV2 and CRN, via Gα, to trigger a signaling process that ultimately restricts the expression domain of WUS. FCP1 interacts with FEA3 to repress WUS from below. Magenta lettering, CLE peptides; blue lettering, receptors, and receptor-like proteins. **(B)** Names of orthologous proteins in Arabidopsis, rice, and maize.

In *Arabidopsis*, meristem size is controlled by the CLAVATA pathway (Miyawaki et al., 2013; Somssich et al., 2016; Bommert and Whipple, 2018; Kitagawa and Jackson, 2019). This pathway is activated by peptide ligands of 12 amino acids processed from the CLE domains of prepropeptides encoded by *CLAVATA3*(*CLV3*)/*Embryo Surrounding Region* (*ESR*)-related genes (i.e., *CLE*) (Djordjevic et al., 2011; Ni et al., 2011). The CLE signal is transmitted by leucine rich repeat (LRR) receptor kinases and acts to restrict the expression domain of transcription factors such as *WUSCHEL* (*WUS*) and *WUSCHEL-related homeobox* (*WOX*) genes (Figure 1A). *Arabidopsis* CLV3 controls the size of all shoot meristems, including the vegetative shoot apical meristem (SAM, for producing leaves), inflorescence meristem (IM, for producing inflorescence branches and flowers) and floral meristem (FM, formed from IM for producing floral organs). CLV3 functions through a signaling pathway that involves LRR receptors including CLAVATA1 (CLV1) and BARELY ANY MERISTEM1-3 (BAM1-3) and others (Nimchuk et al., 2015; Somssich et al., 2016), and the WUS transcription factor, among others (Dodueva et al., 2011; Miyawaki et al., 2013; Kitagawa and Jackson, 2019; and references therein; Figure 1A).

Pathways similar to the Arabidopsis CLAVATA pathway have been found in all plants investigated to date, but details differ. Specifically, the type of meristem (vegetative shoot, inflorescence, branch, flower) regulated by each CLE/CLV/WOX combination varies, as do patterns of redundancy and co-regulation. Recently, Rodríguez-Leal et al. (2019) have analyzed comparisons of Arabidopsis, tomato, and maize CLE knockouts, and shown that compensation mechanisms differ considerably among the three species, depending in part on how paralogous genes are regulated in each species. Because of this unexpected diversity, it is hard to generalize what the specific developmental role of any particular CLE protein might be, and to what extent its function is partially or wholly redundant to that of other CLEs.

Previous work using both phylogenetic analysis and cluster analysis in species of Poaceae has identified CLE proteins in rice, maize and Setaria that are similar to Arabidopsis CLV3 (Je et al., 2016; Goad et al., 2017) (Figure 1B). These include FLORAL ORGAN NUMBER 2/4 in rice (OsFON2/4) and CLE7 (GRMZM2G372364) and CLE14 (AC191109.3_FG001) in maize (Je et al., 2016; Goad et al., 2017). The grass genes form a clade/cluster (called group 1D3 by Goad et al., 2017), indicating that all are more closely related to each other than any is to CLV3 and predicting that their function should be similar to each other. At the same time, the grass genes in cluster 1D3 are more closely related to CLV3 than to any other CLEs (Je et al., 2016).

Unlike in Arabidopsis, the function of CLV3 orthologs in grasses appears restricted to a subset of shoot meristems, although whether this is due to tissue specificity or gene redundancy in some organs is less clear. Mutations in the rice gene Os*FON2* affect only the FM, which becomes larger and produces more floral organs (Suzaki et al., 2006), whereas the IM and BM are apparently not affected. In contrast, *Zmcle7* mutants produce an enlarged IM (Rodríguez-Leal et al., 2019), suggesting that the particular meristems controlled by OsFON2 and ZmCLE7 are different. This apparent functional differentiation is also seen in the receptor proteins. OsFON1, which is similar to CLV1, interacts with OsFON2 in the FM in rice (Chu et al., 2006; Suzaki et al., 2006). Conversely, the maize LRR receptor THICK TASSEL DWARF (TD1, similar to CLV1) functions only in the IM and FM, without significantly affecting the vegetative SAM, although the developmental role of the gene might differ in a different genetic background (Bommert et al., 2005; Kitagawa and Jackson, 2019).

Because the presumed CLV3 orthologs in rice and maize appear to control only particular meristems, other CLE proteins must be involved in specifying meristem size in the inflorescence, branches and flowers. One attractive candidate is FON2 CLE PEPTIDE-RELATED1 (FCP1), which is similar to FON2 in sequence and appears closely related (Je et al., 2016; Goad et al., 2017). In maize, *ZmCLE7* and *ZmFCP1* are the only CLE genes to be significantly upregulated in *cle7* mutants in the ear (female inflorescence), and when mutated, produce an enlarged inflorescence meristem (Rodríguez-Leal et al., 2019). However, FCP1 functions in a pathway parallel to CLE7/FON2 rather than being fully redundant (Je et al., 2018; Kitagawa and Jackson, 2019) (Figure 1A); the effects of mutations are additive in double mutants. ZmCLE7 interacts genetically with TD1 (similar to CLV1; Figure 1B) and ZmFCP1 with FEA3 receptors; both peptides also likely signal through FASCIATED EAR 2 (FEA2, similar to CLV2), but then signals are transmitted by different downstream effectors, CORYNE or CT2 (Je et al., 2016, 2018).

Having data only from rice and maize makes it difficult to generalize results to other grasses and cereal crops. Other species could have developmental networks and functions similar to those in rice, or in maize, or may use some novel combination of ligands and receptors. One possibility is that rice and maize are each characteristic of their own subfamilies (Oryzoideae and Panicoideae, respectively). If this is true, then the results for maize should also apply to other panicoid crops such as sorghum, foxtail millet, pearl millet, barnyard millet, and proso millet. We have investigated this possibility by studying green millet, *Setaria viridis*, the wild progenitor of foxtail millet (*S. italica*) (Le Thierry d'Ennequin et al., 2000; Benabdelmouna et al., 2001; Fukunaga et al., 2006; Hunt et al., 2008) and a tractable model system (Brutnell et al., 2010; Yang et al., 2018; Zhu et al., 2018; Mamidi et al., 2020). In their comprehensive survey of CLE genes in seed plants, Goad et al. (2017) searched the genome of *S. viridis* using a model-based search tool (HMMER3; http://hmmer.org) and identified 41 CLE proteins based on sequence similarity and presence of an identifiable CLE domain. These proteins were then clustered into groups with similar sequences. Only one *S. viridis* gene clustered with (i.e., appeared similar to) *OsFON2*; here we call this locus *SvFON2*.

In the current study we have disrupted *SvFON2* and discovered a strong effect on the inflorescence meristem, similar to mutants of the co-orthologs in maize, but little or no effect on floral meristems, contrasting with *Osfon2* (Suzaki et al., 2006) and suggesting a panicoid genetic network. We then investigated the *SvFON2* co-expression network to predict possible signaling components. Our work identifies a *CLE* gene whose most obvious function is to control the inflorescence and branch meristems in panicoid grasses; we also provide new evidence for *CLE* gene diversification among grass subfamilies, and highlight possible targets of this peptide ligand.

## Materials and Methods

### Plant Material

All data presented here are from *Setaria viridis*, using either accession A10.1 or ME034v (hereafter A10 and ME034 for simplicity). Published data on inflorescence development and gene expression are based on the accession A10, which is a line that has been used for years for *S. viridis* genetics studies. A full genome sequence was released in 2012 (v1.1; Bennetzen et al., 2012) and updated and substantially improved in 2020 (v2.1; Mamidi et al., 2020). The line ME034, originally collected by Matt Estep (now at Appalachian State Univ.) in Manitoba, has come into common use recently because it is easier to transform. It was used here for CRISPR-Cas9 modification of the target gene, *SvFON2*. A genome sequence has recently been released for this accession (Thielen et al., 2020), although it was not available when most of the work reported here was conducted. The two accessions of *S. viridis* are morphologically similar, although development of ME034 is generally more rapid than that of A10 (Kellogg, pers. obs.).

### Histology, Scanning Electron Microscopy (SEM), and *in situ* Hybridization

Scanning electron microscopy was performed as described in Zhu et al. (2018) for A10 plants. For ME034 wildtype and mutant plants, inflorescences were hand-dissected from *S. viridis* seedlings at 12, 14, and 17 days after sowing (DAS). Samples were then dehydrated, critical point dried using a Tousimis Samdri-780a, mounted on stubs and sputter coated using a Tousimis Samsputter-2a. Images were taken with a Zeiss Evo 10 scanning electron microscope at 20 kV at the biology department of Washington University in St. Louis.

For histology, tissues surrounding the shoot apical meristem or inflorescence primordia from 6, 9, and 12 DAS ME034 seedlings were fixed, dehydrated, paraffinized, embedded, and sectioned as described in Hodge and Kellogg (2016). Sections were then deparaffinized in 100% Histoclear twice, 10 min each, rehydrated through an ethanol series (100, 100, 90, 70, 50, 0%, 2 min each step), and stained with 0.05% (w/v) toluidine blue for 1 min. After staining, slides were rinsed and dehydrated by dipping into water, 95 and 100% ethanol three times, 10 dippings each, followed by two xylene washes, 5 min each. Samples were then mounted in Permount with a coverslip and imaged using a Lecia DM 750 microscope at 10–40X.

mRNA *in situ* hybridization was conducted to characterize the expression pattern of *SvFON2* using fixed and embedded developing inflorescences. The primers CTTGCGTTGCTGGTTCATC and AAGGTGTGATCGGCTGCT were used to prepare the probes and *in situ* hybridization followed a previously-described protocol (Jackson et al., 1994).

### Expression and Genomic Region of *SvFON2*

A complete list of *S. viridis CLE* genes was retrieved from Goad et al. (2017), which used the *S. viridis* A10 genome v1.1. We verified that all gene locus names but one were unchanged in version 2.1 of the genome (Mamidi et al., 2020); we updated the one old name (Sevir.J013000) to its new name (Sevir.3G041960) for this paper. Also *SvFCP1* was not annotated in *S. viridis* v1.1 but was annotated in v2.1 (Sevir.7G142950) so we have included that name as well and retrieved the relevant expression data from the raw reads provided by Zhu et al. (2018). Expression, quantified in Transcripts per Million (TPM), of all 42 *CLE* genes in early inflorescence development [10, 12, 14, 15, 16, 18 days after sowing (DAS)] was extracted from the comprehensive RNA-seq data reported for A10 in Zhu et al. (2018) and is reported in Supplementary Table 1. *CLEs* with zero TPM throughout inflorescence development in all six developmental stages are defined as non-expressed genes. For data display and ease of visualization, we considered genes for which at least one sample had TPM > 6. We did not display data for Sevir.3G184200 in which all values were zero except for replicate 4 at 15DAS, which had 6.72 TPM.

Grass *FON2* genomic regions were compared using the GEvo function of CoGe (Lyons and Freeling, 2008; Lyons et al., 2008; Tang et al., 2011) (https://genomevolution.org/coge/). Sequences from *Brachypodium distachyon* (Bd21; JGI v2.0), *Oryza sativa japonica* (Phytozome 11 v323), *Setaria viridis* (A10, Phytozome v2.1), and *Zea mays* [B73, Phytozome 10 (via Gramene) vRef_Gen_v3 and MaizeSequence.org v1] were used for comparison. All genome sequences were unmasked. The *BdFCP1* gene is present but not annotated in the genome of *Brachypodium distachyon* line Bd21 (v2.0); therefore, its closest neighboring genes were used to infer the genomic context for CoGe analysis.

### CRISPR-Cas9 Cloning and Tissue Culture Transformation

To generate mutants in *SvFON2*, CRISPR-Cas9 gene editing technology was used. Constructs were assembled as described (Cermák et al., 2017). Guide RNAs (gRNAs) were designed near protospacer adjacent motif (PAM, required for gRNA targeting) sites using websites http://crispor.tefor.net and http://crispr.hzau.edu.cn/CRISPR2/ (Liu H. et al., 2017). The two gRNAs, ACCACCACGGCAGCCCGTGG (gRNA1) and ACCGCCAAATGATCCACCGC (gRNA2), targeted the exonic region flanking the CLE domain and were predicted to have high specificity. The two guide RNAs were assembled into the module 2 vector pMOD_B2518 via the Esp3I/BsmBI cloning site and module 3 vector pMOD_C2616 via the BsaI cloning site, respectively. Then, the module 1 vector pMOD_A1110 containing Cas9 and hygromycin phosphotransferase genes, module 2 and module 3 vectors were assembled into a destination vector pTRANS_250d via the AarI cloning site. Constructs were sequenced to verify they were error-free and the assembled destination vector was transformed into the Agrobacterium strain AGL1.

Tissue culture transformation was performed as described in Brutnell et al. (2010). Callus was initiated from embryos of sterilized *S. viridis* ME034 seeds, incubated with Agrobacterium suspension, and selected with 40 mg/l hygromycin. Callus was subsequently transferred to plant regenerative medium to grow shoots for transgenic lines and then to rooting medium to recover roots under constant selection of 20 mg/l hygromycin. Transgenic plants were then transferred to soil for genotyping, phenotyping and seed harvest.

### Plant Growth, Transgenic Plant Screening, and Phenotyping

*Setaria viridis* ME034 plants were grown in a greenhouse with conditions described in Acharya et al. (2017); previous work had found that the line ME034 was much healthier and produced more seeds when grown in the greenhouse compared to our controlled environment growth chamber. For root phenotypes, *S. viridis* plants were grown in germination pouches as described in Acharya et al. (2017).

DNA was extracted from leaves using methods described in Edwards et al. (1991). T0 plants were screened by PCR targeting Sevir.2G209800, a gene encoding a C2H2-type zinc finger protein in the *S. viridis* genome as a positive control (primers CAGCAAGCCGCCTATATGGAG and TCGTCTCAGGAGTGGCCAAGT), and the hygromycin phosphotransferase gene on the transgene fragment (primers AGGCTCTCGATGAGCTGATGCTTT and AGCTGCATCATCGAAATTGCCGTC). Editing rate was calculated by edited gene copies divided by total available gene copies. In the T1 generation, the same PCR primers were used to screen for stable lines in which the transgene (Cas9 and the hygromycin selectable marker) was segregated out to prevent any further undesired editing. The *SvFON2* gene region was amplified using primers GCTGGTTCATCCAGTGCAGG and TCAAGGTGTGATCGGCTGCTC and the PCR product was sequenced to screen for edited lines in T0 and then confirmed again in T1 stable lines. Stable and homozygous T2 or T3 lines were used for phenotyping.

Head out date was recorded around 16–21 days when at least half of the panicle emerged from the leaf sheath. Other phenotypes were measured when the plants were 4 weeks old unless otherwise indicated. Plant height and panicle length were measured using a ruler. The third and sixth leaves were cut at the tip to aid leaf number counting. The number of primary inflorescence branches/cm and the number of spikelets per primary branch were counted from a 1 cm region that was 1 cm above the lowermost branch of the main panicle. Experiments were repeated at least three times with at least five plants (and generally more) for each genotype in each replicate. Statistical significance was examined by Welch's two sample *t-*test as implemented in R, testing whether the difference in means between genotypes was significantly different from zero.

### Co-expression Network Analysis for *SvFON2*

To plot the *SvFON2* co-expression network, the “skyblue module” containing *SvFON2* from the Weighted Gene Co-expression Network Analysis (WGCNA) data in Zhu et al. (2018) was used. In these WGCNA data, a weight value was assigned to the connection (edge) between two genes (node). After filtering by weight > 0.185, connections between known genes for inflorescence development and the genes that they directly connected with were selected to construct a co-expression network in Cytoscape v3.4.10.

### LRR Receptors and *WUS/WOX* Homologs

LRR receptors and *WUS/WOX* genes in *S. viridis* were identified using blastp, (https://blast.ncbi.nlm.nih.gov/) with genes reported in Lian et al. (2014) and Liu P. L. et al. (2017), respectively, as bait. Genes were further curated by domain search and annotation from Biomart of EnsemblPlants (http://plants.ensembl.org, accessed on July 1st, 2017). Expression of these genes during early inflorescence development was also extracted from data reported in Zhu et al. (2018).

## Results

### *SvFON2* Is Expressed in Early Inflorescence Development

**Figure 2:**
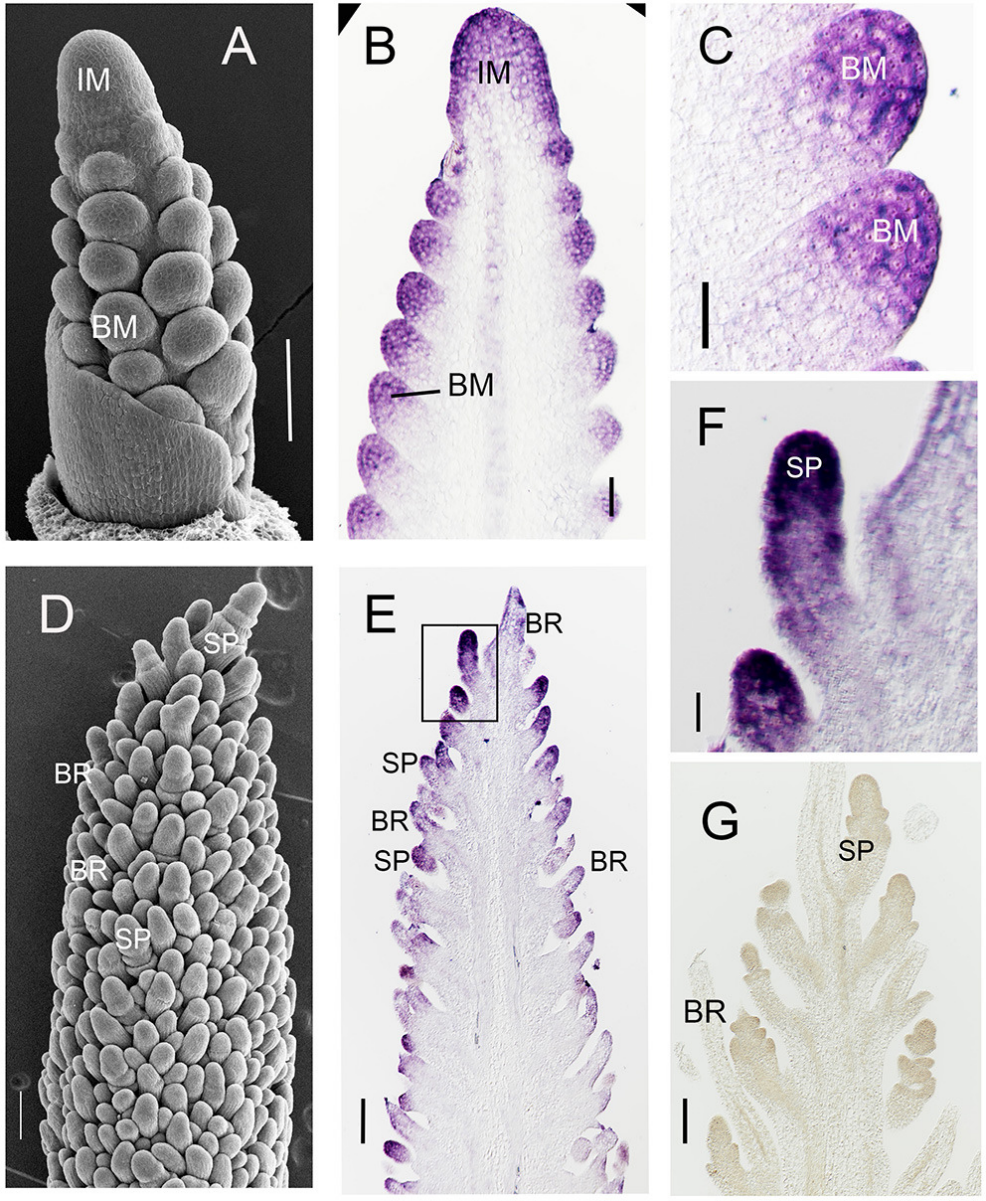
SvFON2 is expressed in early inflorescence, branch, and spikelet meristems. SEM, showing structure of the inflorescence. Sectioned inflorescences from comparable stages showing *SvFON2* expression in the inflorescence meristem, branch meristems, spikelet meristems. **(A,D)** SEM images from early development of A10; images by Matt Box. **(B,C,E–G)** Expression of *SvFON2, in situ* hybridization, ME034. **(B)** inflorescence with branch meristems, slightly more mature than that in **(A)**. **(C)** branch meristems. **(D–F)** inflorescence with differentiated spikelets and bristles; ridges visible on spikelets are glumes. **(F)** close-up of area inside box in **(E)**. **(G)** sense control. Scale bars: **(A–E,G)**, 100 μm; **(F)**, 30 μm. IM, inflorescence meristem, BM, branch meristem, SP, spikelet, BR, bristle.

As a first step to understand the function of *SvFON2* in inflorescence development, we used *in situ* hybridization with a gene-specific probe for *SvFON2*, and considered the stage at which the inflorescence and branch meristems were specified (ca. 11 days after sowing, DAS, Figure 2A) and a slightly later stage when spikelets were clearly formed and floral meristems initiated (Figure 2D). Species of *Setaria* also produce sterile branches known as bristles in their inflorescences; the bristles lose their meristems during the course of development (Doust and Kellogg, 2002). Each spikelet is associated with one or more bristles. *SvFON2* was clearly expressed in inflorescence meristem and branch meristems at 11 DAS (Figures 2B,C). Expression was not confined to particular layers or zones of the meristem but rather extended through the apex of the inflorescence and branch. *SvFON2* was also expressed in spikelet meristems (Figures 2E,F). *SvFON2* expression appeared lower in developing bristles, and expression was not detected in the sense control (Figure 2G).

To capture the dynamics of *SvFON2* expression throughout inflorescence development, we retrieved data for all CLE genes from an available gene expression resource that summarizes gene expression from IM initiation to floral organ development (Zhu et al., 2018). Expression of *SvFON2* (Sevir.8G183800) was low at 10 DAS, increased as branch meristems initiated (12 DAS) and transitioned to spikelet meristems (14 DAS), with values mostly between 5 and 10 TPM through 16 DAS and then dropping to near its original levels as floral meristems differentiated (18 DAS; Supplementary Figure 1 and Supplementary Table 1). In contrast, *SvFCP1* expression was generally zero to 1 TPM until 15 DAS, when it briefly exceeded 5 or 6 TPM, and then dropped back to near zero. This is consistent with previous findings that *ZmFCP1* is expressed in leaf primordia and may control SAM maintenance non-cell-autonomously (Je et al., 2016), so its expression in studies such as ours might be expected to be low. Sequence comparisons indicate that FCP1 gene sequences are conserved among grasses (Supplementary Figure 2B), consistent with the hypothesis that the function of SvFCP1 is similar to that of ZmFCP1 and are likely not central to grass inflorescence development.

In addition to *SvFON2* and *SvFCP1*, 21 other Setaria CLE genes were expressed in early inflorescence development, with TPM > 5 for at least one individual value (Supplementary Table 1). Of this set of 23 CLE genes, we display the 18 most highly expressed (Supplementary Figure 1; raw data, means and standard deviations in Supplementary Table 1). Other than *SvFON2* and *SvFCP1*, only a couple have well-characterized functions in any angiosperm. Goad et al. (2017) assigned all *CLE* genes in plants to one of 12 groups (1A−1J, 2, and 3) based on sequence similarity, with *FON2/CLV3*-like and *FCP1* genes falling into Group 1D. The other genes expressed in Setaria inflorescences were mainly assigned to groups 1B, 1C, 1E, and 1J, all of unknown function despite representation throughout the angiosperms. In particular, three genes from subgroup 1E1 had higher expression than *SvFON2* (Sevir.3G191400, Sevir.9G131200, Sevir.9G048500), especially at early developmental stages (Supplementary Figure 1).

The most highly expressed *CLE* (Sevir.1G367300) is a member of group 2 (Supplementary Figure 1) that functions in vascular development (Goad et al., 2017). Arabidopsis proteins in this group inhibit differentiation of tracheary elements thereby regulating xylem formation (Hirakawa and Bowman, 2015); they also appear to function in axillary bud formation (Yaginuma et al., 2011). Related CLE genes appear to be highly conserved among seed plants and thus Sevir.1G367300 may also be involved in vascular differentiation in *S. viridis* inflorescence development.

### *SvFON2* Regulates the Inflorescence Meristem

**Figure 3:**
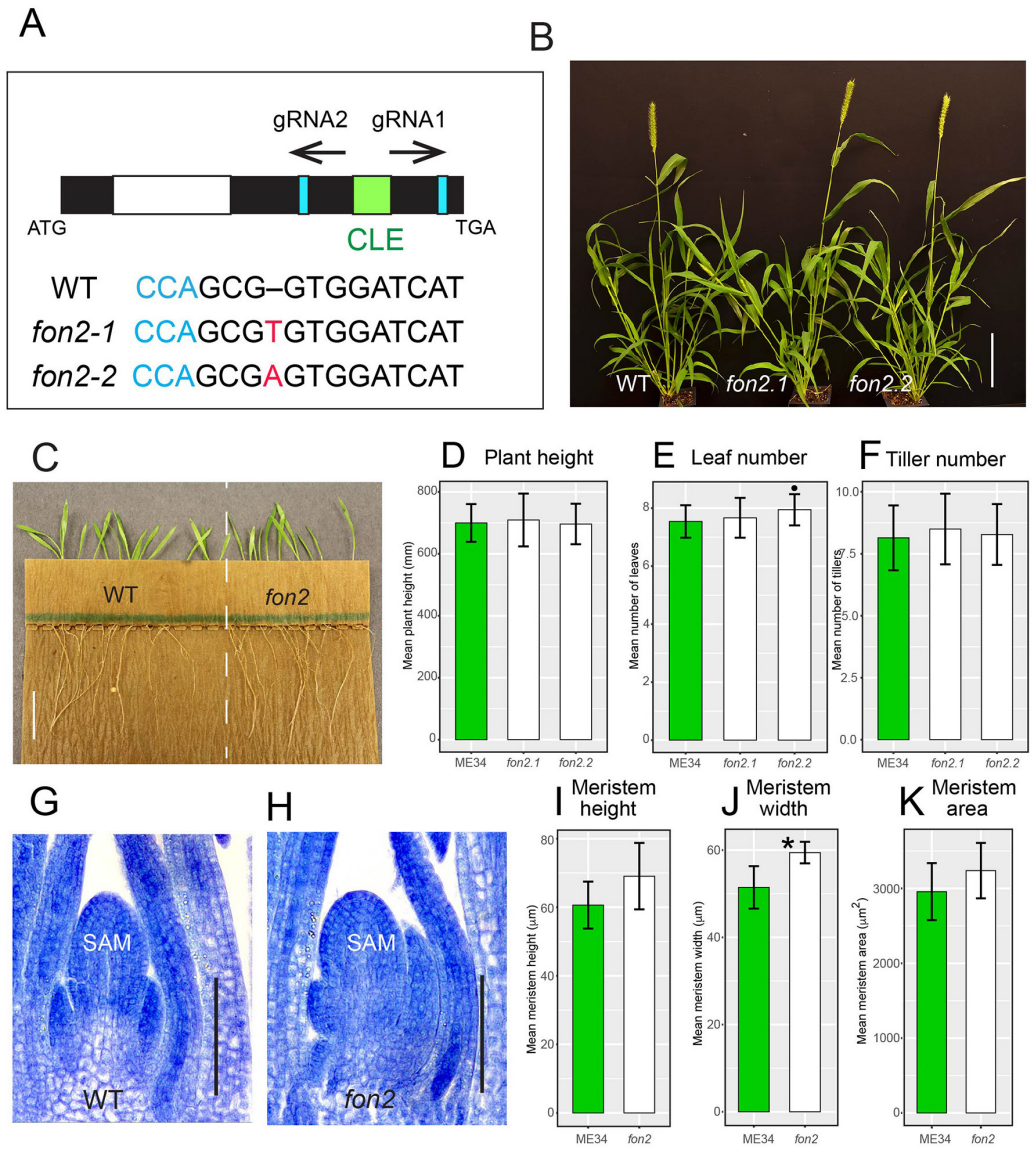
*S. viridis fon2* mutants and phenotypic characterization during vegetative development. **(A)** Gene structure of *SvFON2* and gene editing in the mutants. Black, white, and green colored boxes represent exon, intron, and CLE domain, respectively, of the *SvFON2* gene. Blue boxes and text show PAM sites. Black arrows indicate target sites for the two guide RNAs (gRNA1 and gRNA2). ATG and TGA indicate the start and stop codons of the gene. Sequence below the gene model shows sequence comparison among wildtype (WT) and two independently edited mutants (*fon2-1* and *fon2-2*). Red highlighted bases indicate the 1 bp insertion in the gRNA2 target region. Representative set of 4-week old plants **(B)**. Roots from 5 DAS WT and *fon2* showing no significant growth defects **(C)**. Plant height **(D)**, leaf number **(E)**, and tiller number **(F)** of WT (green bars) and *fon2* mutants (*fon2-1* and *fon2-2*; white bars). Toluidine blue stained median sections of shoot apical meristems (SAM) at 6 DAS for WT **(G)** and *Svfon2* mutant **(H)**. SAM height **(I)**, width **(J)**, and area **(K)** of WT and *fon2* vegetative meristems at six DAS. Significance values by Welch's *t-*test: *p < 0.01. Numbers of replicates for each statistical comparison listed in Table 1. Error bars are ± one standard deviation. SAM, shoot apical meristem. Scale bar: **(B)**, 10 cm; **(C)**, 2 cm; **(G,H)**, 50 μm.

To further test the function of *SvFON2* during inflorescence development, we knocked it out using CRISPR-Cas9 gene editing (Cermák et al., 2017). Preliminary screening of seven T0 transgenic plants showed editing only at the gRNA2 target sites, and four plants contained editing in at least one of the two copies of *SvFON2*, for an editing rate of 29%. Further genotyping in T1 of these four transgenic lines retrieved two differently edited *fon2* alleles. Stable homozygous lines were obtained for these two alleles, designated as *Svfon2-1* and *Svfon2-2*, and were used for analysis. *Svfon2-1* and *Svfon2-2* each had a one base pair (T and A, respectively) insertion just after the G nucleotide at CDS position 103 (Figure 3A). This insertion disrupts the reading frame after residue 34 (position 47 in the alignment in Supplementary Figure 2A), upstream of the CLE domain. Since the active product from a CLE gene is the processed CLE domain (Djordjevic et al., 2011; Ni et al., 2011), these mutations are likely to disrupt the function of *SvFON2* completely.

**Table 1A:** Mean ± one standard deviation for plant traits comparing wild type (WT, ME034v), *Svfon2.1* and *Svfon2*.2.

	**WT mean value**	***Svfon2.1* mean value**	***Svfon2.2* mean value**	**p, WT vs. *Svfon2.1***	**p, WT vs. *Svfon2.2***	**p, *Svfon2.1* vs. *Svfon2.2***
Plant height (mm)	700.00 ± 65.55 (34)	709.28 ± 85.26 (18)	696.5 ± 65.27 (18)	0.6845	0.8512	0.6171
Leaf number	7.54 ± 0.55 (35)	7.67 ± 0.69 (18)	7.94 ± 0.54 (18)	0.5141	**0.01586**	0.1862
Tiller number	8.14 ± 1.31 (35)	8.5 ± 1.42 (18)	8.28 ± 1.23 (18)	0.3811	0.7132	0.6194
Days to heading	19.03 ± 0.71 (35)	17.78 ± 0.94 (18)	18.22 ± 0.43 (18)	**3.376e-05**	**4.381e-06**	0.0812
Panicle length (mm)	78.46 ± 12.05 (34)	86.17 ± 9.81 (18)	84.89 ± 16.15 (18)	**0.01554**	0.1461	0.7763
Branches per cm	10.91 ± 3.67 (17)	19.12 ± 6.62 (13)	15.13 ± 4.49 (12)	**0.000834**	**0.01413**	0.09007
Spikelets per primary branch	11.40 ± 2.67 (10)	9.00 ± 1.35 (5)	9.87 ± 1.44 (5)	**0.03825**	0.174	0.3567

*p-values of Welch's two-sample t-tests test whether the difference in means is significantly different from 0*.

**Figure 4:**
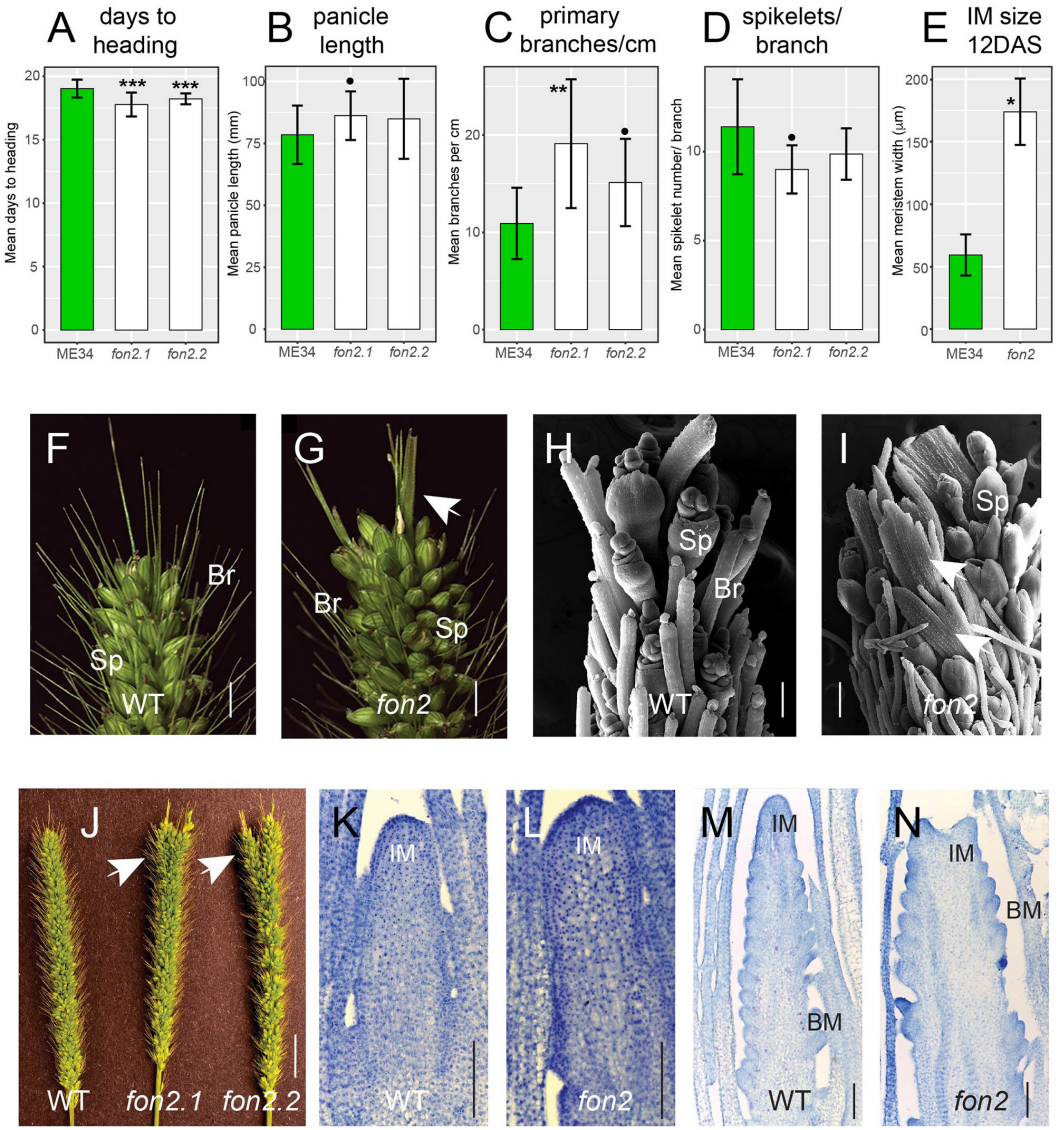
Phenotypic characterization of *fon2* during reproductive development. Days to heading **(A)**, panicle length **(B)**, primary branches/cm **(C)**, spikelets/branch **(D)** of WT (green bars) and *fon2* mutants (*fon2-1* and *fon2-2*; white bars). **(F–I)** Panicle apices from WT **(F,H)** and *Svfon2* **(G,I)**. **(H,I)** Representative SEM images of 17 DAS WT **(H)** and *Svfon2* mutant **(I)**. White arrows show novel sheet-like structures in the mutant. Main panicles **(J)** from WT and mutants. White arrows show that the mutant panicle apices are larger and have more tips. **(K–N)** Toluidine blue stained median sections of inflorescence meristems at nine DAS **(K,L)** and 12 DAS **(M,N)**. **(K,M)**, WT; **(L,N)**, *Svfon2*. Significance values by Welch's *t-*test: *p < 0.01; **p < 0.001; ***p < 0.0001; black dot, *p* < 0.05. Numbers of replicates for each statistical comparison listed in Table 1. Error bars are ± one standard deviation. IM, inflorescence meristem; BM, branch meristem; Sp, spikelet; Br, bristle (sterile branch). Scale bars: **(F,G)**, 5 mm; **(H,I)**, 100 μm; **(J)**, 1 cm; **(K–N)**, 100 μm.

*Values significant at p < 0.05 marked in boldface. Sample sizes in parentheses. See also Figures 3, 4*.

Neither mutant allele affects overall plant growth characteristics. Vegetative characteristics of the two *Svfon2* alleles did not differ significantly from each other or from WT in the number of leaves, plant height, and tiller number (Figures 3B,D–F; Table 1A). Roots of *Svfon2* mutants also grew similarly to WT (Figure 3C), suggesting that the root apical meristem was not affected in *Svfon2* mutants. Because of the similarity between the two *Svfon2* alleles, hereafter we refer to them together as *Svfon2*.

Despite the overall similarity of vegetative characteristics, *Svfon2* mutant plants exhibited subtle differences in the size of the SAM at 6 DAS, before its transition to IM (Figures 3G,H). Width, height and area of the SAM in *SvFON2* were about 15%, 13% and 9% larger than WT, respectively (Figures 3I–K; Table 1B). However, the difference was only significant for meristem width (*p* = 0.00183; two-tailed *t*-test).

**Table 1B:** Mean ± one standard deviation for plant traits comparing wild type (WT, ME034v) to *Svfon2* mutants.

	**WT mean value**	***Svfon2* mean value**	**p, WT vs. *Svfon2***
6 DAS meristem height (μm)	60.631 ± 6.85 (8)	69.078 ± 9.71 (9)	0.05492
6 DAS meristem width (μm)	51.458 ± 4.86 (8)	59.430 ± 2.47 (9)	**0.00183**
6 DAS meristem area (μm^2^)	2,957.42 ± 340.52 (5)	3,238.22 ± 331.09 (5)	0.271
12 DAS meristem width (μm)	59.254 ± 16.5 (5)	173.893 ± 26.5 (3)	**0.0070**
Seed weight	986.82 ± 155.61 (22)	823.25 ± 150.99 (15)	**0.00322**

*p-values of Welch's two-sample t-tests test whether the difference in means is significantly different from 0*.

*Values significant at p < 0.05 marked in boldface. Sample sizes in parentheses*.

Reproductive aspects of *Svfon2* differed significantly from WT. Inflorescences in the *Svfon2* mutants emerged from the sheath (headed out) about 1 day earlier than WT (Figure 4A), suggesting that the mutants transitioned from vegetative to reproductive growth earlier. Although overall panicle length was not significantly different between *Svfon2.2* mutant plants and WT and only weakly significant between *Svfon2.1* and WT (Figure 4B, Table 1A), primary branch number per cm (i.e., branch density) was significantly higher in *Svfon2* (Figure 4C, Table 1A). The number of spikelets per branch was somewhat lower in the mutants, but the values were only significant in the comparison of *Svfon2.1* and WT (Figure 4D, Table 1A).

The most striking phenotype of *Svfon2* was in the panicle tips (Figures 4F–I). Abnormal apices were observed in all mutant panicles including the main inflorescences and those from the tillers. The panicle tips of *Svfon2* were larger than WT, often divided into two or more parts, and contained novel structures that were neither spikelets nor bristles (Figures 4G,I,J). Scanning electron microscopy (SEM) of the inflorescence tips showed that terminal sheet-like structures produced prickle hairs, characteristic of bristles (Figures 4H,I).

To verify that mutant phenotypes were caused by the mutation in *SvFON2*, we crossed *Svfon2-1* to WT plants. The F1 plants had normal panicles, and nine out of 36 plants had larger panicle tips (25%) in the F2, indicating a single recessive mutation. Genotyping the segregating F2 plants showed complete linkage between the homozygous mutation in *SvFON2* and the larger-tip panicle phenotype, suggesting that the *SvFON2* mutation was indeed responsible for the mutant phenotype.

**Figure 5:**
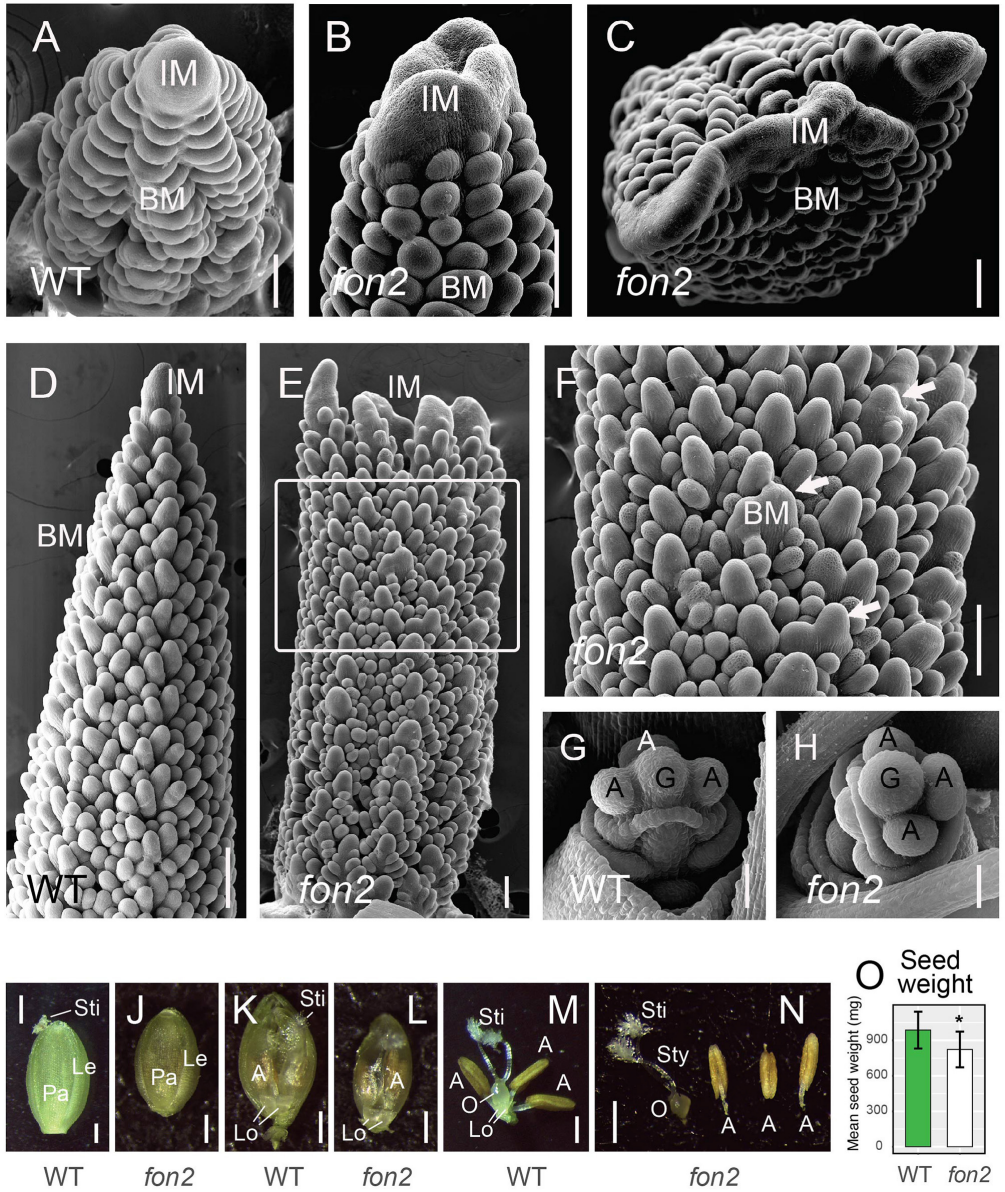
Inflorescence meristems and early floral development of *Svfon2* mutants. **(A–F)** Representative scanning electron microscope images of 12 DAS WT **(A,D)** and *Svfon2* mutants **(B,C,E,F)**. **(F)** corresponds to the area marked by a white box in **(E)**. White arrows show enlarged primary branch meristems. **(G,H)** Representative scanning electron microscope images of 17 DAS WT **(G)** and *Svfon2* mutant **(H)**. **(I–N)** Florets of WT **(I,K,M)** and *Svfon2* mutant **(J,L,N)** immediately before anthesis. WT in **(I,M)**, A10; WT in **(K)**, ME34. **(I,J)** Upper (fertile) floret, lemma, and palea, viewed from the adaxial (relative to the floral axis) side. **(K,L)** Glumes and lemmas removed, floret viewed from the abaxial (relative to the floral axis) side, with the floral parts lying on the palea, and partially enveloped by the hyaline margins of the palea. Two normally formed lodicules are clearly visible, and the golden brown anthers can be seen through palea margins. The feathery stigma is slightly exserted from the WT palea, whereas it is still enclosed in the *Svfon2* image. **(M,N)** Floral organs, showing gynoecium with an ovary, two styles and two stigmas, and three anthers on the right. **(O)** Total seed weight in the main panicle of WT (green bar) and *svfon2* (white bar). Significance values by Welch's *t-*test: *p < 0.01. Numbers of replicates for each statistical comparison listed in Table 1. Error bars are ± one standard deviation. IM, inflorescence meristem; BM, branch meristem; A, anther; G, gynoecium; Le, upper lemma; Lo, lodicule; O, ovary; Pa, upper palea; Sti, stigma; Sty, style. Scale bars: **(A–H)**, 50 μm; **(I–N)**, 0.5 mm.

The abnormal panicle tip phenotype in *Svfon2* suggested a defect in IM control. While IMs of WT inflorescences were more or less conical with clearly organized rows (Figures 4K,M, 5A,D), the IM in *Svfon2* mutants was about three times wider than WT by 12 DAS (Figures 4E,N; Table 1B) and formed abnormal shapes (Figures 4L,N, 5B,C,E,F). We looked at many more SEM photos and longitudinal sections than indicated by the sample size for IM width reported in Table 1B, and in all cases we observed considerable difference in size between WT and mutant meristems. However, because of the angle of the SEMs and the fact that the mutant meristems are highly irregular in shape, we could only obtain precise numbers for a small number of samples for which we were confident of our measurements. Nonetheless, the difference was significant (*p* = 0.0070), reflecting the clear distinction in size. Because of a larger IM, more branch meristems were initiated in *Svfon2*, consistent with our observations that the mutants had more primary panicle branches per cm (Figure 4C, Table 1A). Primary branch meristems were also larger than in WT, but not as markedly so as the IM (Figures 5E,F). The slightly reduced number of spikelets per primary branch in the mutants (Figure 4D) suggested that the *Svfon2* mutation may have disrupted higher order branching and/or spikelet development. Perhaps because the mutants had fewer spikelets per primary branch and shorter time to flowering, overall seed weight in the main panicle was significantly lower in mutant plants (*p* = 0.00322; Table 1B, Figure 5O).

### *SvFON2* Mutations Have No Effect on Floral Organ Number or Morphology

Mutants of the closest homolog of *SvFON2* in rice, *Osfon2*, produced extra anthers and pistils in the flowers (Suzaki et al., 2006). To study whether *Svfon2* gave rise to similar phenotypes, we counted floral organs in *Svfon2*, including the glume, lemma, lodicule, palea, ovary, stigma, and anther. Unlike *Osfon2*, the number and shape of floral organs in *Svfon2* appeared entirely normal (Figures 5G–N, Table 2), indicating that SvFON2 is not the major CLE peptide controlling the floral meristem.

**Table 2:** Numbers of floral organs in WT and *svfon2* mutant flowers.

	**Glume**	**Lemma**	**Lodicule**	**Upper Palea**	**Ovary**	**Stigma**	**Anther**
WT	2	2	2	1	1	2	3
*fon2*	2	2	2	1	1	2	3

*N > 20*.

We cannot rule out the possibility that floral meristems in *Svfon2* mutants are slightly larger or smaller than in WT. Floral meristem size in *S. viridis* is difficult to quantify. The inflorescence has five or six orders of branching and thus bears flowers in many different orientations and stages of development. The precise orientation of the section on any particular floral meristem is not easily controlled to obtain a perfectly medial section; because sections are often more or less oblique, measurements are highly inaccurate. A similar problem exists for SEM photos. The images in Figures 5G–N hint that mutant flowers might in fact be smaller than in WT, which would also be consistent with the reduced seed weight per panicle, although a full exploration of this possibility would require extensive measurements of spikelets and floral organs during development. Nonetheless, we are confident that if meristem size and organ number were increased as markedly as in rice *FON2* mutants, we would have detected the change.

### *SvFON2* Co-expression Network and Its Signaling Components

To identify genes that have a similar expression pattern to that of *SvFON2* and thus potentially identify new factors for inflorescence meristem size determination in *S. viridis* and other panicoid grasses, we defined the *SvFON2* co-expression network using expression data from the IM developmental time series published by Zhu et al. (2018) (Supplementary Figures 3, 4; Supplementary Table 2). RNA-seq data were collected in quadruplicate for inflorescences at 10, 12, 14, 15, 16, and 18 DAS, as noted above. The 10 DAS time point captures the IM soon after the transition from the vegetative SAM and the subsequent days capture branching, spikelet formation, and early floral development. For clarity of display, this network was filtered to show only the 26 genes whose expression is most tightly correlated with that of *SvFON2* (weight > 0.185); this set is called “FON2-network” in Supplementary Table 2. As expected, the genes in this network showed a pattern nearly identical to that of *SvFON2*, in which expression is low at 10 DAS, increases to 14 or 15 DAS, remains high through 16 DAS and then drops substantially by 18 DAS (Supplementary Figure 4). Not surprisingly, the FON2-network included orthologs of *Fasciated ear 4* (*Fea4*, encoding a bZIP transcription factor), and *Ramosa3* (*Ra3*, encoding a trehalose-phosphate phosphatase), genes encoding negative regulators that control IM size and branch initiation without affecting floral organs (Satoh-Nagasawa et al., 2006; Pautler et al., 2015). The *SvFON2* network also contained an ortholog of *Rough sheath1* (*Rs1*) which encodes a class I KNOX homeodomain transcription factor and is important for meristem control and lateral organ initiation (Schneeberger et al., 1995). Identification of known developmental genes for IM control in the *SvFON2* co-expression network is consistent with our finding that *SvFON2* also functions in IM size.

*SvFON2* expression is strongly correlated with that of such uncharacterized genes as Sevir.9G527000 and Sevir.5G100000 (Supplementary Figures 3, 4), which exhibit moderate expression during early inflorescence development (Supplementary Table 2). These genes contain no annotated domains, and BLASTP searches against plant genomes in Phytozome and against GenBank find no genes of known function. In addition, none of the BLAST hits aligns with more than 64% of the query sequence. These two loci are therefore interesting candidates for future characterization.

The *SvFON2* network also contains five F-box domain-containing genes and one gene encoding a RING-type E3 ligase (Supplementary Figures 3, 4). F-box proteins and E3 ligases are core components of the ubiquitin ligase complex that is responsible for protein degradation, with the F-box protein providing substrate specificity (Sharma et al., 2016). F-box genes can also function in hormone regulation and transcriptional activation (Lippman et al., 2008). Several transcription factors including a zinc finger protein and MADS-box proteins are also in the *SvFON2* network (Supplementary Figures 3, 4). Function of these genes and their genetic relationships remain to be investigated.

In addition to the 21 genes in the tightly correlated FON2-network, 264 additional genes had expression values correlated with those of *SvFON2* but at much lower levels (Supplementary Table 2, “FON2_edge_allgenes”). Among these less strongly connected genes were several that were annotated as LRR receptors or *WUS/WOX* genes, as expected in a CLE-gene network. These are shown in Supplementary Figure 3 as linked to *SvFON2* with dotted lines to indicate that they were added to the diagram manually and are not as tightly connected as the other genes shown in the figure. Five LRR receptor genes were co-expressed with *SvFON2* (Supplementary Figure 3, Supplementary Tables 2, 3), one of which is homologous to *CLV1/TD1* (Sevir.4G294000/*SvTD1*). Other LRR receptor genes that are co-expressed with *SvFON2* include homologs of EXCESS MICROSPOROCYTES1 (EMS1) which functions in somatic and reproductive cell fates in the *Arabidopsis* anther (Zhao et al., 2002; Li et al., 2017) and HAESA-LIKE 1 (HSL1) that regulates floral organ abscission in *Arabidopsis* (Jinn et al., 2000; Gubert and Liljegren, 2014) (Supplementary Table 2). One WUS/WOX gene (Sevir.5G266300) was also co-expressed with *SvFON2* (Supplementary Figure 3, Supplementary Table 2). The function of the products of these genes in *S. viridis* inflorescence meristems remains to be determined.

We expected that the *SvFON2* co-expression network would include only a subset of the LRR receptors and *WUS/WOX* transcription factors that were expressed during early inflorescence development. Using sequence BLAST and domain searches, we identified homologs of these genes in *S. viridis*. As expected, the LRR receptor family is large, with at least 438 genes in *S. viridis* (Supplementary Table 3). Using data from Zhu et al. (2018), we found 247 LRR receptor genes were expressed in early inflorescence development with a collective TPM greater than two across all six stages (Supplementary Table 3). Among them were homologs of LRRs that function in the Arabidopsis CLV3 signaling pathway, including *CLV1/TD1, CLV2/FEA2, FEA3* (Je et al., 2016, 2018), *BARELY ANY MERISTEM 1-3* (*BAM1-3*) (DeYoung et al., 2006), *ERECTA* (*ER*) (Mandel et al., 2014), *CORYNE* (*CRN*) (Muller et al., 2008), *RECEPTOR-LIKE PROTEIN KINASE 2* (*RPK2*) (Kinoshita et al., 2010), and *CLAVATA3 INSENSITIVE RECEPTOR KINASES* (*CIKs*) (Hu et al., 2018; Xu and Jackson, 2018). Expression of these genes was moderate to high (Supplementary Figure 5, Supplementary Table 3), with *ER* and *BAM* homologs exhibiting the highest and relatively stable expression during these stages (Supplementary Figure 5, Supplementary Table 3). Several other LRR receptors are also highly expressed, such as Sevir.3G424200, during early inflorescence development (Supplementary Table 3), but their roles are unknown in *S. viridis* or other species and thus they are interesting candidate genes for future studies.

Similar analyses found 12 *WUS/WOX* genes in *S. viridis* (Supplementary Figure 5, Supplementary Table 3), seven of which were expressed in early inflorescences with a collective TPM greater than two. Homologs of *WOX3, WOX13*, and *WOX9* had relatively high expression during early inflorescence development (Supplementary Table 3).

## Discussion

### *S. viridis* FON2 Controls Inflorescence Meristem but Not Floral Meristem Development

Using two independently edited alleles generated by CRISPR-Cas9 technology, we showed that SvFON2 plays a prominent role in IM size control in Setaria, as the co-orthologous protein ZmCLE7 does in maize (Rodríguez-Leal et al., 2019).

Although the basic framework of the signaling pathway involving a CLE ligand and LRR receptors appears conserved among eudicots and monocots, the developmental roles of individual proteins differ. SvFON2 has its major developmental role in the IM but not the FM, different from OsFON2 which specifically regulates the FM without affecting the IM, suggesting that the developmental role of this subgroup of CLEs has become further differentiated between Panicoideae and Oryzoideae. Because the *Svfon2* mutant had normal floral organs, other proteins, likely other CLEs, must control FM and floral organ development in Setaria. *Svfon2* also had a largely normal SAM, again pointing to other CLEs regulating that meristem.

Divergence in the developmental roles of the FON2 CLEs could be caused by differences in (a) gene expression, (b) post-translational modifications, (c) patterns of redundancy and dosage compensation mechanisms, or some combination of the three. In contrast to FCP1 whose genomic regions in different grass species have high degree of collinearity (Supplementary Figure 6), the genomic regions encompassing the FON2 orthologs are not perfectly collinear (Supplementary Figure 7), suggesting that the regulatory environment may not be conserved for FON2 genes. However, *OsFON2* is expressed in the SAM, IM and FM even though the mutant phenotype is only seen in the FM (Suzaki et al., 2006). Similarly, *SvFON2* is expressed in a broad spatiotemporal window from IM initiation through to FM and floral organ development but only the IM is substantially altered in the *Svfon2* mutant. Thus, gene regulation alone is unlikely to explain the divergence in developmental roles for this group of CLEs.

Another possibility for *CLE* divergence lies in peptide modifications and interactions with downstream factors. Differences in the processing of the pre-propeptides, amino acid sequences in the CLE domain, and the level and type of modifications (e.g., arabinosylation) of CLE peptides can change their activity and binding affinity to receptors (Ohyama et al., 2009; Xu et al., 2015). CLE domains in grass *FON2* genes differ in several amino acids (Supplementary Figure 2), which could affect the number and nature of post-translational modifications.

Third, different degrees of pathway redundancy in different tissues also contribute to divergence. Some tissues are controlled by a single CLE peptide and its pathway, so this CLE plays a predominant role, whereas other tissues are controlled by multiple pathways, so the role of each CLE can be often masked by others (Je et al., 2018; Rodríguez-Leal et al., 2019). All of these factors together constitute a complex yet delicate network controlling IM and FM, which is critical to understand the morphological diversity of grass inflorescences.

### Other CLE Genes Function During Inflorescence Development

A total of 23 out of 42 *CLEs* were expressed during early inflorescence development in *S. viridi*s, but only a few of these have been studied in other grasses. The rice FON2 SPARE1 (FOS1) protein functions in FM control. The functional allele of this gene in *indica* varieties acts as a suppressor of *fon2* allele (Suzaki et al., 2009); however, no knock-out of this gene has been reported. The two *S. viridis FOS1* homologs [Sevir.1G144900 (*SvFOS1B*) and Sevir.3G241500 (*SvFOS1A*)] are scarcely expressed in early inflorescence development, even at 18 DAS when most floral meristems should have initiated (TPM collectively from six stages < 0.5) (Supplementary Table 1). Thus, FOS1A and FOS1B might have different roles in Setaria than their co-orthologue does in rice. Likewise, overexpression and peptide application studies implicate the rice protein FCP2 in FM and/or IM control (Suzaki et al., 2008; Ohmori et al., 2014), whereas Chu et al. (2013) suggest that rice FCP2 is involved in root meristem maintenance and metaxylem development. *SvFCP2* (Sevir.4G139000) is not expressed at all during early *S. viridis* inflorescence development (Supplementary Table 1). Whether the protein functions differently in rice and Setaria is therefore unclear, since no studies of rice or Setaria *FCP2* genes provide evidence from loss of function mutants or responses in mutants lacking putative receptors. With small ligands such as the CLEs, peptide application experiments are hard to interpret, because they may or may not reflect interactions in normal development.

In contrast, several other *CLEs* are far more highly expressed in inflorescences than *SvFON2* (Supplementary Figure 1), making it interesting to test their function in inflorescence development in the future. These *CLEs* could also regulate the FM and/or vegetative SAM, as discussed above, but equally likely could be involved in other aspects of inflorescence development.

The genomic context of the CLE genes in grasses has also received scant attention. We show here that while FCP1 orthologs appear in generally collinear regions in several species of grasses (Supplementary Figure 6), the region surrounding FON2 is apparently more dynamic (Supplementary Figure 7). Whether such local rearrangements affect gene regulation is unknown. The comparative genomic positions of CLE genes in general have not been analyzed, perhaps in part because they are short genes and not always well-annotated.

### *SvFON2* Co-expression Network and Its Signaling Components

For additional insight into the role of *SvFON2* in signaling, we extracted the *SvFON2* co-expression modules from our published analysis of gene expression during inflorescence development (Zhu et al., 2018). *SvFON2* expression is correlated with expression of the Setaria orthologs of *Fasciated Ear 4* (*SvFEA4*), *Ramosa3* (*SvRA3*), and *RoughSheath1* (*SvRS1*) (Supplementary Figures 3, 4), whose homologs in maize all regulate meristem maintenance or determinacy (Schneeberger et al., 1995; Satoh-Nagasawa et al., 2006; Pautler et al., 2015). In maize, the CLAVATA pathway that involves the FEA2 and FEA3 receptors controls meristem homeostasis (Je et al., 2016, 2018), and FEA4 also negatively controls meristem maintenance, independently of the CLAVATA pathway (Pautler et al., 2015). Since FEA2 function is, at least partially, mediated by *CLE7* (the closest homolog of *SvFON2* and *OsFON2* in maize) (Je et al., 2018), it is possible that *SvFON2* functions in parallel to *SvFEA4* in *S. viridis*, although this remains to be experimentally tested. The genetic relationship between *SvFON2* and *SvRA3* and *SvRS1* has not been explored, and it would be interesting to examine this in *S. viridis* or maize.

The *SvFON2* co-expression network includes a number of F-box containing genes and E3 ligase genes (Supplementary Figures 3, 4). F-box containing genes that function in meristem control and/or inflorescence architecture include *ABERRANT PANICLE ORGANIZATION 1* (*APO1*) (Ikeda et al., 2007) and *LARGER PANICLE* (*LP*) (Li et al., 2011) from rice, *UNUSUAL FLORAL ORGANS* (UFO) from *Arabidopsis* and its ortholog *ANANTHA* (*AN*) from tomato (Lippman et al., 2008), and several F-box genes in wheat (Hong et al., 2012). Similarly, E3 ligases such as *SHOOT APICAL MERISTEM ARREST 1* (*SHA1*) (Sonoda et al., 2007) and *Nicotiana tabacum RING DOMAIN CONTAINING PROTEIN1* (*NtRCP1*) from tobacco (Wang et al., 2015) function in meristem maintenance and/or floral transition. Therefore, we hypothesize that these F-box containing genes and E3 ligases function in meristem regulation during inflorescence development.

Identification of LRR receptors and their expression analyses suggested that homologs of CLV1/TD1, CLV2/FEA2, FEA3, ERECTA (ER), BARELY ANY MERISTEM1-3 (BAM1-3), CORYNE (CRN), RECEPTOR-LIKE PROTEIN KINASE2 (RPK2), and CLAVATA-interacting kinases (CIKs) are expressed in early inflorescences (Supplementary Figures 3, 5, Supplementary Table 3) and could be receptors of SvFON2. Given that LRR receptors and co-receptors interact in a complex way for various functions (Smakowska-Luzan et al., 2018), SvFON2 could signal through multiple receptors. Among the potential receptors, SvTD1 is most likely because TD1 in maize also functions in the inflorescence meristem (Bommert et al., 2005) similar to SvFON2 in *S. viridis*. In support of this, *SvTD1* is one of the few LRR receptor genes whose expression is correlated with that of *SvFON2* (Supplementary Table 2, Supplementary Figure 5).

No WOX protein has been functionally characterized in Setaria. Based on their expression (Supplementary Figure 5, Supplementary Table 3), homologs of *WOX3, WOX13, WOX9*, and *WUS* are candidates for regulation by the *SvFON2* signaling pathway, although *SvWOX9A* (Sevir.5G266300) is most tightly co-expressed with *SvFON2* and is the best candidate for direct regulation (Supplementary Figures 3, 5). *WOX9* (also called *STIMPY*) functions in embryonic patterning and shoot apical meristem maintenance (Wu et al., 2005, 2007) and its ortholog *COMPOUND INFLORESCENCE* from tomato (Lippman et al., 2008; Zheng and Kawabata, 2017) is essential for inflorescence development and architecture, but roles of their homologs in grasses have not been studied. Specific functions of the two *WOX3* homologs in *S. viridis* (Sevir.3G044600/*SvWOX3A* and Sevir.8G020500/*SvWOX3B*) are unknown. *Arabidopsis WOX3* [also called *PRESSED FLOWER* (*PRS*)] and its orthologs in maize, *narrow sheath1* and *narrow sheath2* (*NS1* and *NS2*), are required for lateral sepal development (Matsumoto and Okada, 2001) and/or leaf development (Nardmann et al., 2004) by recruiting organ founder cells from lateral domains of meristems and promoting cell proliferation (Nardmann et al., 2004; van der Graaff et al., 2009). *WOX13* and its paralog *WOX14* in *Arabidopsis* regulate the floral transition, floral organ development and root development (Deveaux et al., 2008). Since *WOX13* and *WOX14* homologs are highly conserved among land plants (Deveaux et al., 2008), their homolog in *S. viridis* (Sevir.5G365700/*SvWOX13*) may play similar roles. WOX4 is regulated by FCP1 in rice (Ohmori et al., 2013) but appears to be mostly involved in maintenance of vegetative meristems; its expression level in *S. viridis* is low (Supplementary Table 3).

### Potential Roles of SvFON2 in Flowering Time and Yield

One unexpected result of this study was a significant acceleration of inflorescence development, with the mutant plants heading out about 1 day earlier than WT. We infer that this reflects acceleration of the transition from the vegetative SAM to an IM, although it may or may not affect time to anthesis. We have not found many reports of a similar effect of mutations in CLV3-like genes in other systems, although a change in meristem size might indirectly influence developmental timing. Much of the work on CLV3-like genes has described loss-of-function mutations, in which the effect on the meristem is so dramatic that timing changes may be difficult to disentangle. However, variation in meristem size is associated with variation in days to flowering in maize (Leiboff et al., 2015) and naturally-occurring alleles in a CLV3-like gene are associated with days to flowering in chickpea (*Cicer arietinum*) (Basu et al., 2019).

Any locus that affects inflorescence meristem size has the potential to affect yield. For example, natural variation among *FEA2* and *FEA3* alleles in maize affects inflorescence meristem size and hence kernel row number and number of grains per ear (Je et al., 2016; Trung et al., 2020). Given this information from maize, we may have expected that yield would be higher when *Svfon2* was disrupted, but it was lower. The observation of fewer spikelets per primary branch may help explain this result, as may subtle size differences in florets. Primary branches in wild type *S. viridis* continue to branch multiple times, producing secondary, tertiary, and even higher order branches (Doust and Kellogg, 2002). We speculate that even modest disruption of the primary branch meristems in *Svfon2* mutants may affect these later rounds of branching, which in turn will affect the relative numbers of bristles and spikelets. In addition, as shown in maize (e.g., Rodríguez-Leal et al., 2019), other CLE loci are likely to be involved in control of other meristems. Therefore, when using *CLE* genes such as *SvFON2* to engineer related crops for increased yield, it may be necessary to finetune its expression or function by generating knockdown or weaker alleles, instead of making knockout alleles. In addition, it may be necessary to manipulate other *CLE* genes other than *SvFON2* alone.

In summary, we found that the developmental roles of *FON2* genes have diversified among grasses, with *OsFON2* affecting floral meristems and *SvFON2* affecting inflorescence and branch meristems, similar to its maize homolog. Thus, the *SvFON2* gene regulatory network may be shared among panicoid grasses, but likely does not extend to oryzoids. Our bioinformatic analyses predicted genes that may be involved in the *SvFON2* co-expression network and signaling pathway, focusing on LRR receptors and WUS/WOX genes. Along with rapidly advancing gene editing and transformation technologies (Zhu et al., 2017), studies on the function and evolution of *CLE* genes and their signaling pathway components will be accelerated in *S. viridis* and other grasses.

## Data Availability Statement

The datasets presented in this study can be found in online repositories. The names of the repository/repositories and accession number(s) can be found in the article/Supplementary Material.

## Author Contributions

EK and CZ designed the project and produced the figures. HZ did the transformation with input from TB. LL did the *in situ* hybridization with input from DJ. All other experiments and data analysis were carried out by CZ and OC. CZ drafted the paper with subsequent comments and review by EK and DJ. All authors read and approved the final manuscript.

## Conflict of Interest

The authors declare that the research was conducted in the absence of any commercial or financial relationships that could be construed as a potential conflict of interest.

## Supplementary Material

Supplementary Figure 1 Expression of CLE genes in *S. viridis* A10 at six developmental stages. Figure includes all but three CLE genes for which at least one stage had TPM > 5 (see all CLE gene in Supplementary Table 1). *SvFON2* has elevated expression at 10–12 days after sowing (DAS); the pattern is distinct from that of most other CLEs. Note different scales of the vertical axis for the lower six panels. Leading “Sevir.” has been omitted from all gene model names to improve clarity. Each gene model name is followed by the name of the sequence-based cluster to which it was assigned by Goad et al. (2017); color of shading behind names indicates genes from the same cluster. Gene expression in Transcripts Per Million (TPM) at six sequential stages was retrieved from Zhu et al. (2018). The six stages represent IM initiation (10 days after sowing; DAS; see Figures 2A–C), primary (12 DAS) and higher order (14 DAS) branch formation, transition to SMs (15 DAS; Figures 2D–F), differentiation of spikelets and bristles (16 DAS), and floral organ development (18 DAS).

Supplementary Figure 2 Comparison of CLE domain sequences from CLV3/FON2 **(A)** and CLE40/FCP1 **(B)** subgroup genes. Genes from *Arabidopsis thaliana* (At) *Brachypodium distachyon* (Bd), *Oryza sativa japonica* (Os), *Setaria viridis* (Sv), and *Zea mays* (Zm) are shown. Vertical line (arrow) indicates position of the CRISPR-Cas9 induced insertions and subsequent disruptions of the reading frame in the two *Svfon2* alleles.

Supplementary Figure 3 *SvFON2* co-expression network during inflorescence development. Correlations among transcripts in the module containing *SvFON2* from Weighted Gene Co-expression Network Analysis (WGCNA), using data extracted from Zhu et al. (2018); graph constructed in Cytoscape v3.4.10. After filtering by weight > 0.185, connections between key genes involved in plant development (yellow) and the genes that they directly connected with were displayed (Supplementary Table 2). “Sevir.” is omitted from each gene ID for clarity. Line length is proportional to the weight between the nodes. Green highlighted genes are transcription factors in the MADS-box (MADS) or zinc finger protein (ZFP) families. Pink-purple highlighted genes encode proteins containing an F-box or E3 ligase and may be involved in protein degradation. Other genes are highlighted in blue (functional annotation available) or gray (no sequence similarity to any other annotated genes). Expression of five additional *LRR* genes and one *WOX* gene also correlates with that of *SvFon2* but at a level below the 0.185 cut-off weight; these were added manually to the figure by dotted lines. See also Supplementary Figure 4 and Supplementary Table 2.

Supplementary Figure 4 Expression of *SvFON2* (upper left) and individual genes in the *SvFON2* co-expression network during inflorescence development. Expression data for genes in colored ovals in Supplementary Figure 3. Values are in TPM at six sequential stages (10–18 DAS) retrieved from Zhu et al. (2018). Error bars are standard deviations. Note substantial differences in the scale of the vertical axes, reflecting differences in overall expression level. See also Supplementary Tables 2, 3.

Supplementary Figure 5 Expression of *SvFON2* and selected LRR receptor and *WUS/WOX* genes during *S. viridis* early inflorescence development. Gene expression data in TPM at six sequential stages (10–18 DAS) retrieved from Zhu et al. (2018). Error bars are standard deviations. Note differences in the scale of the vertical axes, reflecting differences in overall expression level. See also Supplementary Tables 2, 3.

Supplementary Figure 6 Screenshot of the GEvo analysis from CoGe to compare the ca. 50Kb genomic region around the *FCP1* gene. *FCP1* genes are highlighted in red boxes. Sequences from *Brachypodium distachyon, Oryza sativa japonica, Setaria viridis*, and *Zea mays* were used for comparison. Each black rectangle represents one sequence and the dashed line in the middle divides the two strands. Gene models are drawn in green, with UTRs in blue, and introns in gray. Colored blocks above or below gene models are regions of similarity between all pairwise sequence comparisons. The more and/or wider colored blocks are, the more collinear or stable the genomic region is. Some connections are shown but connectors are omitted around FCP1 itself for clarity. BdFCP1_Bradi5g13241 is not annotated in the genome of *Brachypodium distachyon* line Bd21(v2.0) for the CoGe analysis so the closest neighboring gene was used to infer the genomic context. Analysis can be regenerated at https://genomevolution.org/r/1fssg.

Supplementary Figure 7 Screenshot of the GEvo analysis from CoGe to compare the ca. 50 Kb genomic region around the *FON2* gene. *FON2* genes are highlighted in red boxes. Sequences from *Brachypodium distachyon, Oryza sativa japonica, Setaria viridis*, and *Zea mays* were used for comparison. Each black rectangle represents one sequence and the dashed line in the middle divides the two strands. Gene models are drawn in green, with UTRs in blue and introns in gray. Colored blocks above or below gene models are regions of similarity between all pairwise sequence comparisons. The more and/or wider colored blocks are, the more collinear or stable the genomic region is. Some connections are shown but connectors are omitted around FON2 itself for clarity. Analysis can be regenerated at https://genomevolution.org/r/1fss7.

Supplementary Table 1 **(Sheet 1)** Expression of genes in the SvFon2(Sevir.8G183800) network with weight cutoff at 0.185, genes in Transcripts per Million (TPM) at six different stages of inflorescence development. Weight is a value quantifying the co-expression level of a gene pair generated by Weighted Gene Co-expression Network Analysis (WGCNA) in Zhu et al. (2018). For annotation, WikiGene.description and InterPro.description are from Biomart of EnsemblPlants. Best.hit.arabi.name, arabi.symbol, arabi.defline, Best.hit.rice.name and rice.defline are from the annotation file for the S. viridis genome (v1.1) from Phytozome v12.1. **(Sheet 2)** Expression of all genes directly associated with SvFon2 (Sevir.8G183800) in Transcripts per Million (TPM) at six different stages of inflorescence development. Genes were arranged by weight value quantifying the co-expression level of a gene pair generated by Weighted Gene Co-expression Network Analysis (WGCNA) in Zhu et al. (2018). For annotation, Short_name and Short_description are from classic maize genes. WikiGene.description and InterPro.description are from Biomart of EnsemblPlants. Best.hit.arabi.name, arabi.symbol, arabi.defline, Best.hit.rice.name and rice.defline are from the annotation file for the S. viridis genome (v1.1) from Phytozome v12.1.

Supplementary Table 2 **(Sheet 1)** Expression of genes homologous to known leucine rich receptors in Transcripts per Million (TPM) at six different stages of inflorescence development. For annotation, WikiGene.description and InterPro.description are from Biomart of EnsemblPlants. Best.hit.arabi.name, arabi.symbol, arabi.defline, Best.hit.rice.name and rice.defline are from the annotation file for the S. viridis genome (v1.1) from Phytozome v12.1. Genes were arranged by their expression value, highest to lowest for the sum of expression across six stages. **(Sheet 2)** Expression of leucine rich receptors and WOX genes that are coexpressed with SvFon2 (Sevir.8G183800). Weight is a value quantifying the co-expression level of a gene pair generated by Weighted Gene Co-expression Network Analysis (WGCNA) in Zhu et al. (2018). Expression of genes in Transcripts per Million (TPM) at six different stages of inflorescence development. For annotation, WikiGene.description and InterPro.description are from Biomart of EnsemblPlants. Best.hit.arabi.name, arabi.symbol, arabi.defline, Best.hit.rice.name and rice.defline are from the annotation file for the S. viridis genome (v1.1) from Phytozome v12.1. Genes were arranged by their weight value, starting with those whose expression is most closely correlated with SvFON2. **(Sheet 3)** Expression of genes homologous to known leucine rich receptors (LRR) in Transcripts per Million (TPM) at six different stages of inflorescence development. For annotation, WikiGene.description and InterPro.description are from Biomart of EnsemblPlants. Best.hit.arabi.name, arabi.symbol, arabi.defline, Best.hit.rice.name and rice.defline are from the annotation file for the S. viridis genome (v1.1) from Phytozome v12.1. Genes were arranged by their expression value, highest to lowest for the sum of expression across six stages. **(Sheet 4)** Expression of WUS/WOX genes in Transcripts per Million (TPM) at six different stages of inflorescence development. For annotation, WikiGene.description and InterPro.description are from Biomart of EnsemblPlants. Best.hit.arabi.name, arabi.symbol, arabi.defline, Best.hit.rice.name and rice.defline are from the annotation file for the S. viridis genome (v1.1) from Phytozome v12.1. Genes are arranged by their expression value, highest to lowest for the sum of expression across six stages.
