# Supplementary figures and images for: The CLV3 Homolog in Setaria viridis Selectively Controls Inflorescence Meristem Size
Source: Front Plant Sci. 2021 Feb 15;12:636749. doi: 10.3389/fpls.2021.636749 (PMC7917188; doi:10.3389/fpls.2021.636749)

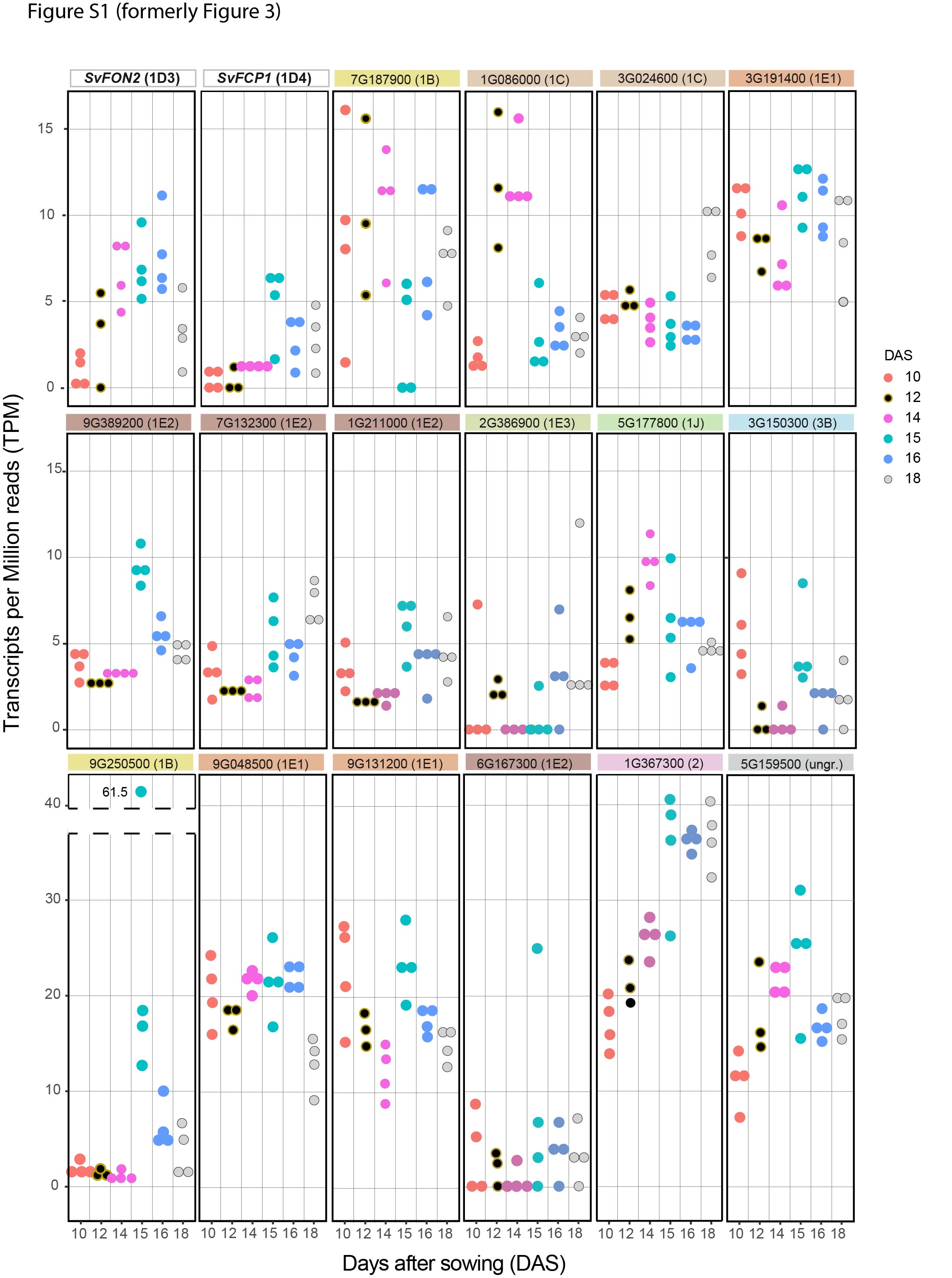

Supplement: Supplementary Figure 1 — Expression of CLE genes in S. viridis A10 at six developmental stages. Figure includes all but three CLE genes for which at least one stage had TPM > 5 (see all CLE gene in Supplementary Table 1). SvFON2 has elevated expression at 10–12 days after sowing (DAS); the pattern is distinct from that of most other CLEs. Note different scales of the vertical axis for the lower six panels. Leading “Sevir.” has been omitted from all gene model names to improve clarity. Each gene model name is followed by the name of the sequence-based cluster to which it was assigned by Goad et al. (2017); color of shading behind names indicates genes from the same cluster. Gene expression in Transcripts Per Million (TPM) at six sequential stages was retrieved from Zhu et al. (2018). The six stages represent IM initiation (10 days after sowing; DAS; see Figures 2A–C), primary (12 DAS) and higher order (14 DAS) branch formation, transition to SMs (15 DAS; Figures 2D–F), differentiation of spikelets and bristles (16 DAS), and floral organ development (18 DAS). [file Image_1.jpeg]

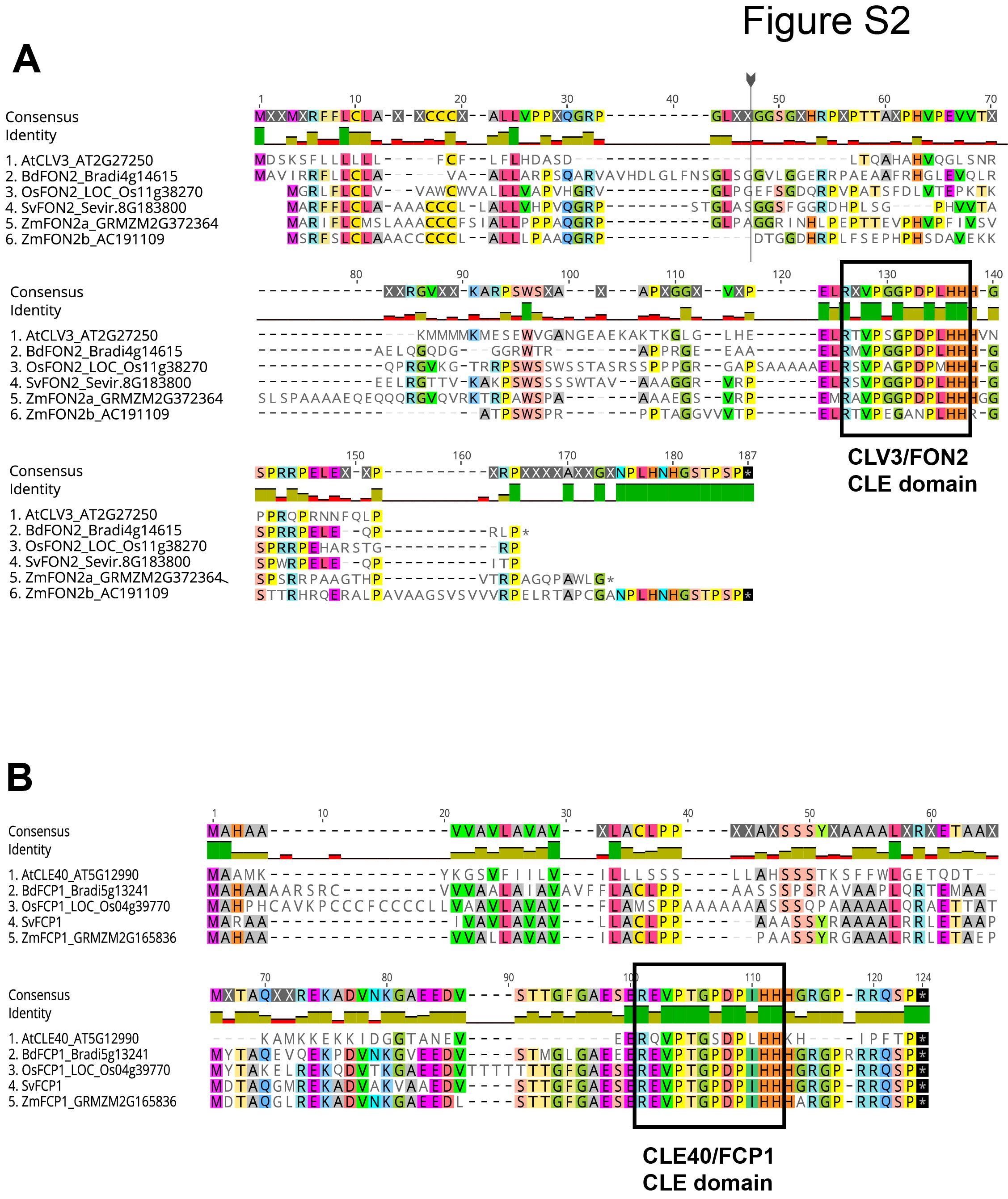

Supplement: Supplementary Figure 2 — Comparison of CLE domain sequences from CLV3/FON2 (A) and CLE40/FCP1 (B) subgroup genes. Genes from Arabidopsis thaliana (At) Brachypodium distachyon (Bd), Oryza sativa japonica (Os), Setaria viridis (Sv), and Zea mays (Zm) are shown. Vertical line (arrow) indicates position of the CRISPR-Cas9 induced insertions and subsequent disruptions of the reading frame in the two Svfon2 alleles. [file Image_2.jpeg]

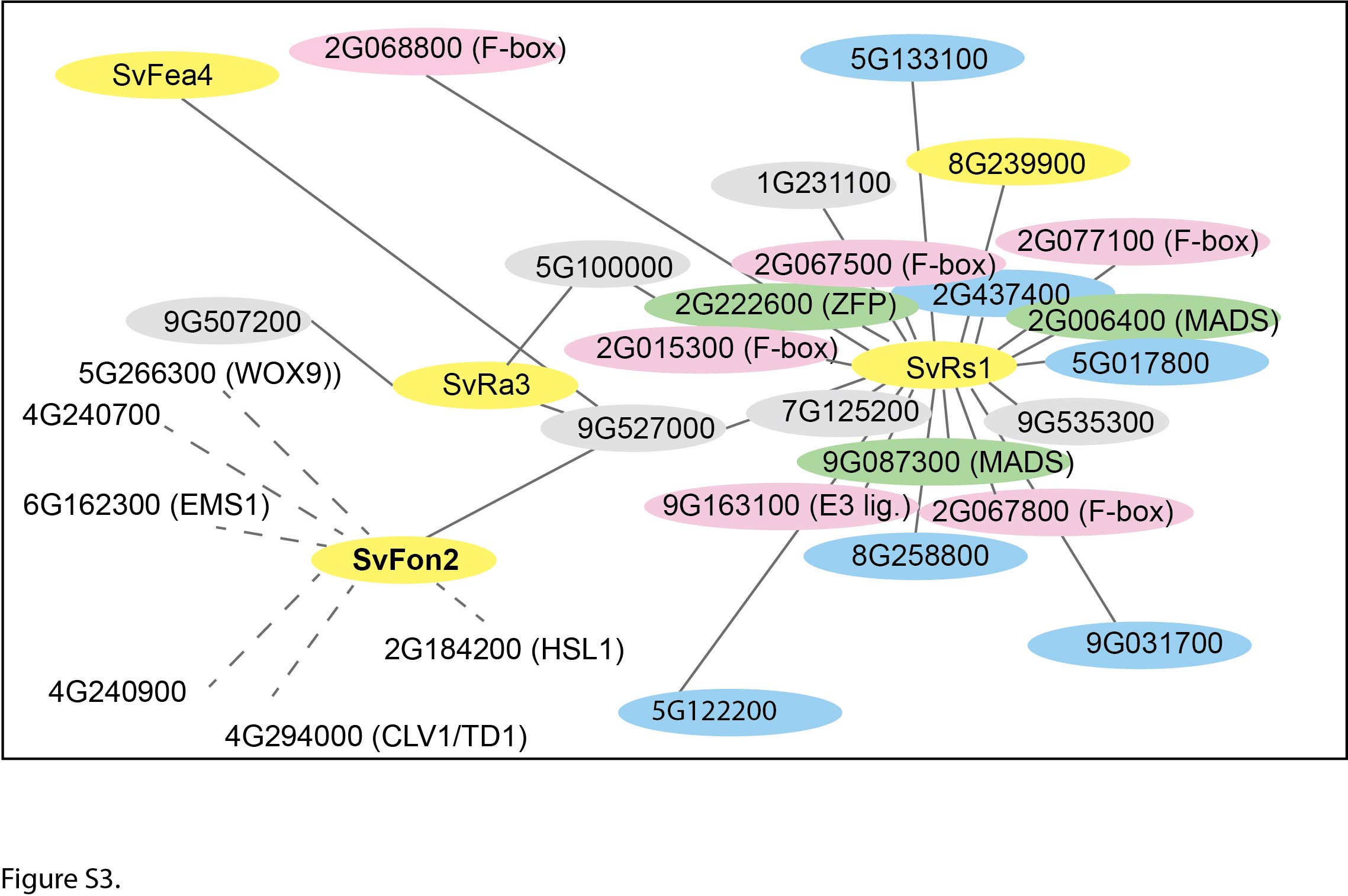

Supplement: Supplementary Figure 3 — SvFON2 co-expression network during inflorescence development. Correlations among transcripts in the module containing SvFON2 from Weighted Gene Co-expression Network Analysis (WGCNA), using data extracted from Zhu et al. (2018); graph constructed in Cytoscape v3.4.10. After filtering by weight > 0.185, connections between key genes involved in plant development (yellow) and the genes that they directly connected with were displayed (Supplementary Table 2). “Sevir.” is omitted from each gene ID for clarity. Line length is proportional to the weight between the nodes. Green highlighted genes are transcription factors in the MADS-box (MADS) or zinc finger protein (ZFP) families. Pink-purple highlighted genes encode proteins containing an F-box or E3 ligase and may be involved in protein degradation. Other genes are highlighted in blue (functional annotation available) or gray (no sequence similarity to any other annotated genes). Expression of five additional LRR genes and one WOX gene also correlates with that of SvFon2 but at a level below the 0.185 cut-off weight; these were added manually to the figure by dotted lines. See also Supplementary Figure 4 and Supplementary Table 2. [file Image_3.jpeg]

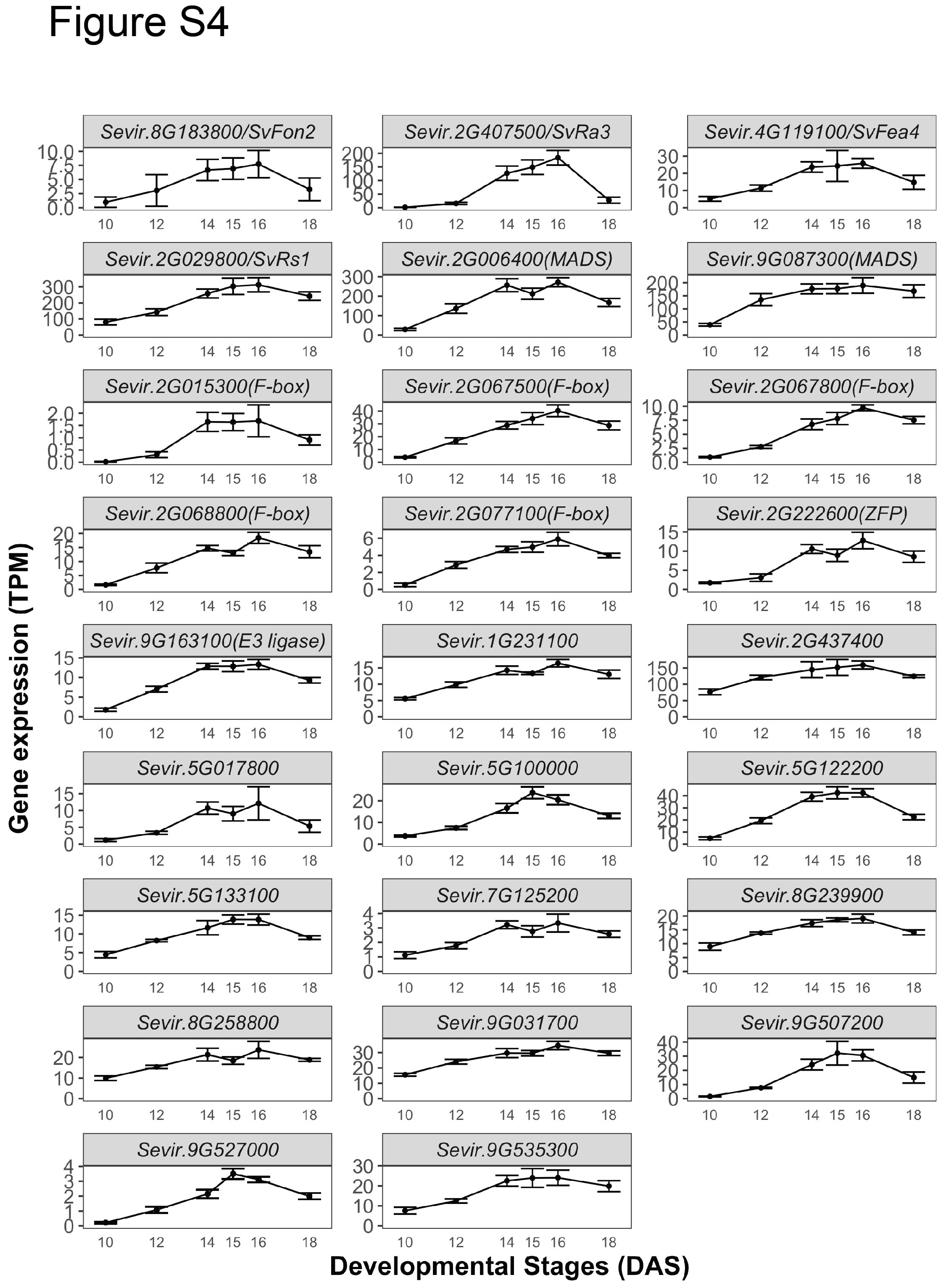

Supplement: Supplementary Figure 4 — Expression of SvFON2 (upper left) and individual genes in the SvFON2 co-expression network during inflorescence development. Expression data for genes in colored ovals in Supplementary Figure 3. Values are in TPM at six sequential stages (10–18 DAS) retrieved from Zhu et al. (2018). Error bars are standard deviations. Note substantial differences in the scale of the vertical axes, reflecting differences in overall expression level. See also Supplementary Tables 2, 3. [file Image_4.jpeg]

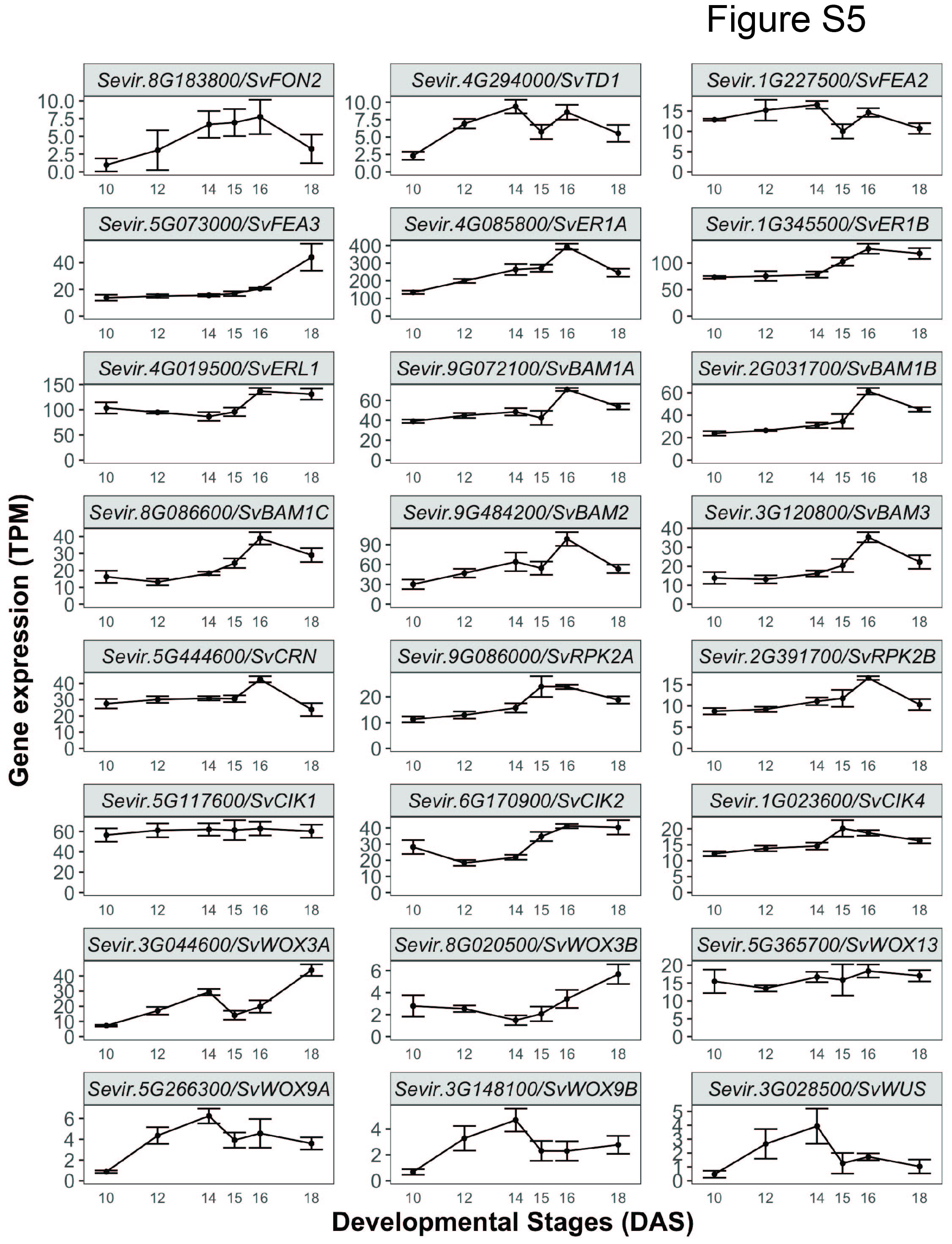

Supplement: Supplementary Figure 5 — Expression of SvFON2 and selected LRR receptor and WUS/WOX genes during S. viridis early inflorescence development. Gene expression data in TPM at six sequential stages (10–18 DAS) retrieved from Zhu et al. (2018). Error bars are standard deviations. Note differences in the scale of the vertical axes, reflecting differences in overall expression level. See also Supplementary Tables 2, 3. [file Image_5.jpeg]

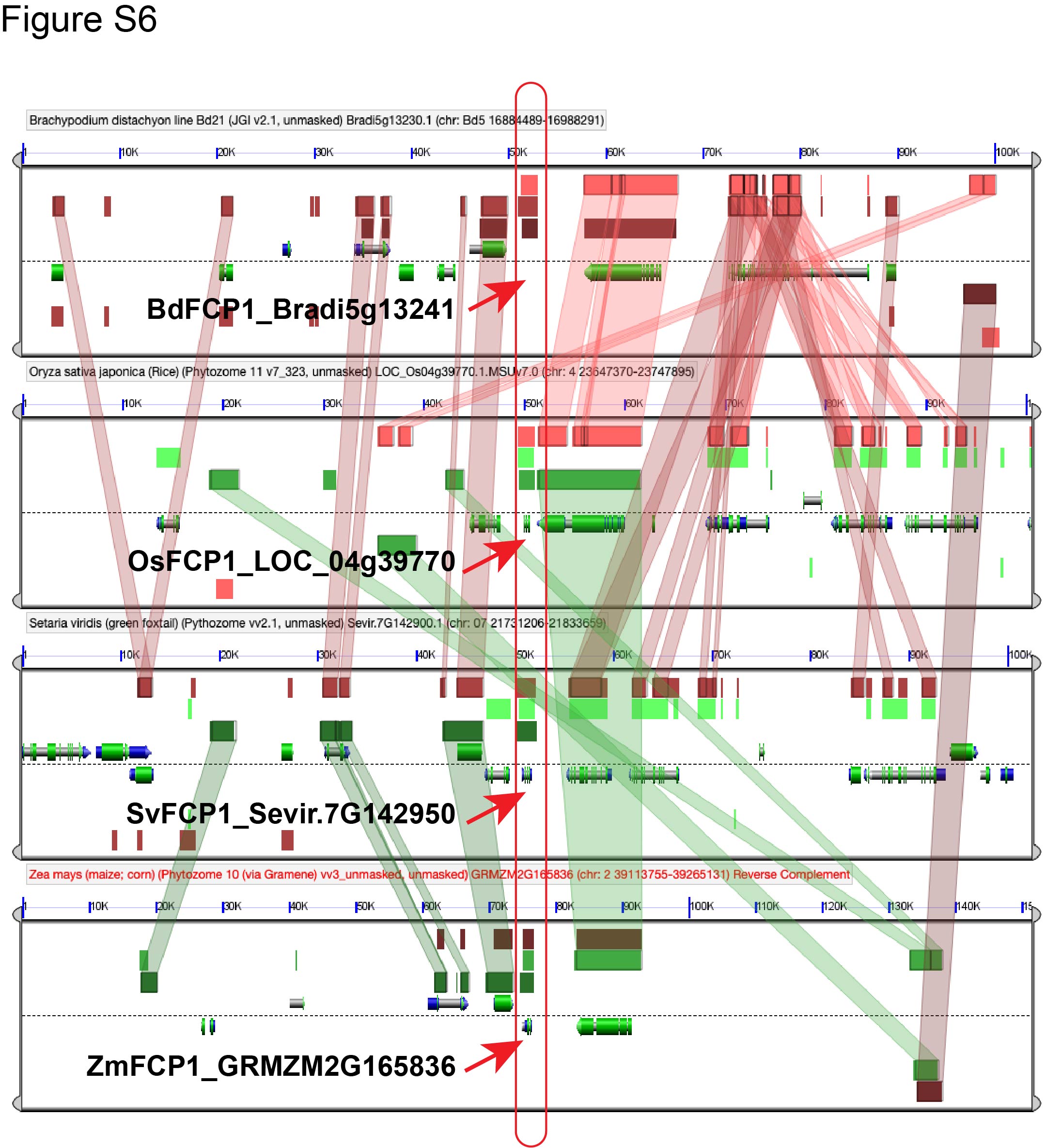

Supplement: Supplementary Figure 6 — Screenshot of the GEvo analysis from CoGe to compare the ca. 50Kb genomic region around the FCP1 gene. FCP1 genes are highlighted in red boxes. Sequences from Brachypodium distachyon, Oryza sativa japonica, Setaria viridis, and Zea mays were used for comparison. Each black rectangle represents one sequence and the dashed line in the middle divides the two strands. Gene models are drawn in green, with UTRs in blue, and introns in gray. Colored blocks above or below gene models are regions of similarity between all pairwise sequence comparisons. The more and/or wider colored blocks are, the more collinear or stable the genomic region is. Some connections are shown but connectors are omitted around FCP1 itself for clarity. BdFCP1_Bradi5g13241 is not annotated in the genome of Brachypodium distachyon line Bd21(v2.0) for the CoGe analysis so the closest neighboring gene was used to infer the genomic context. Analysis can be regenerated at https://genomevolution.org/r/1fssg. [file Image_6.jpeg]

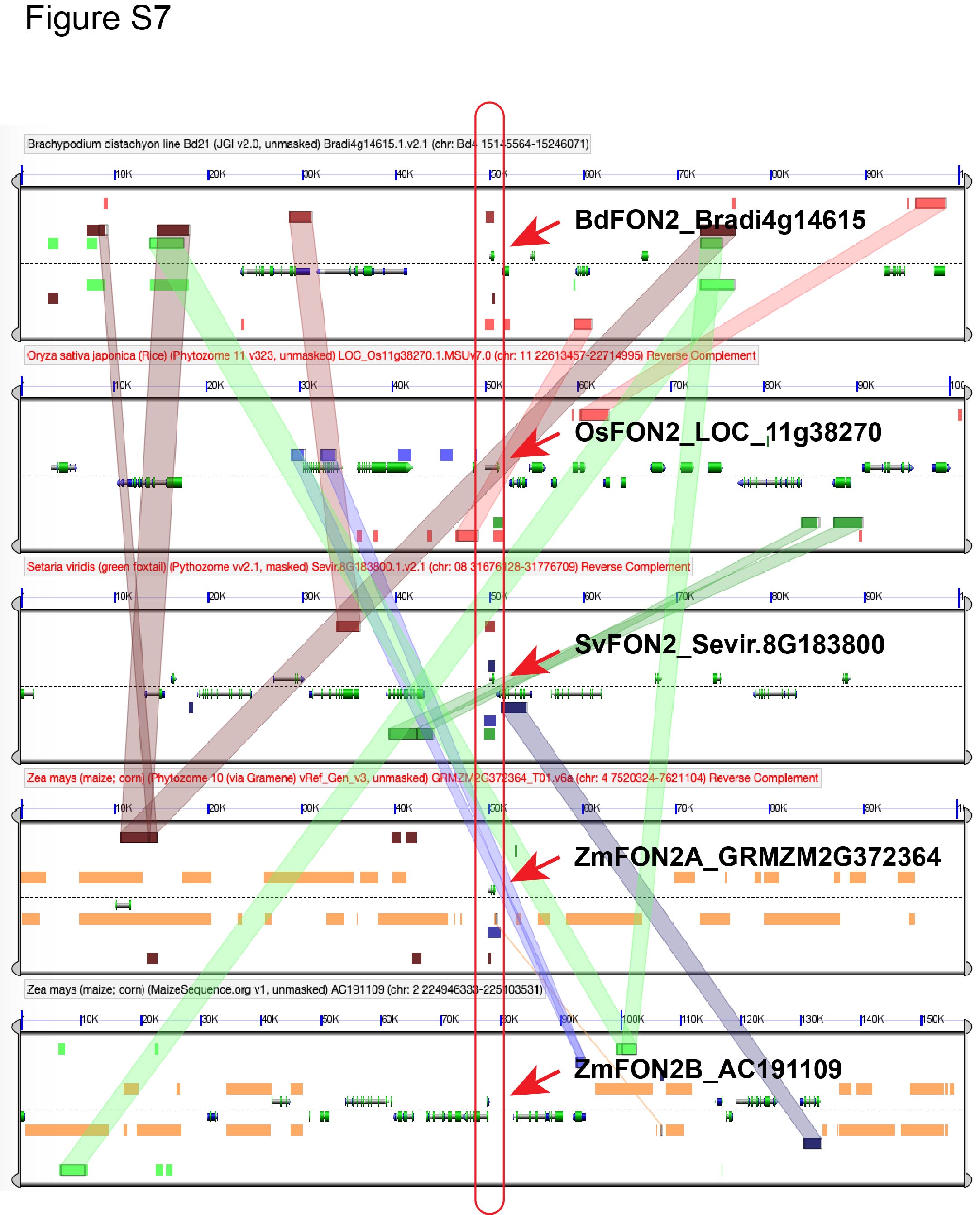

Supplement: Supplementary Figure 7 — Screenshot of the GEvo analysis from CoGe to compare the ca. 50 Kb genomic region around the FON2 gene. FON2 genes are highlighted in red boxes. Sequences from Brachypodium distachyon, Oryza sativa japonica, Setaria viridis, and Zea mays were used for comparison. Each black rectangle represents one sequence and the dashed line in the middle divides the two strands. Gene models are drawn in green, with UTRs in blue and introns in gray. Colored blocks above or below gene models are regions of similarity between all pairwise sequence comparisons. The more and/or wider colored blocks are, the more collinear or stable the genomic region is. Some connections are shown but connectors are omitted around FON2 itself for clarity. Analysis can be regenerated at https://genomevolution.org/r/1fss7. [file Image_7.jpeg]
